# Moringin Induces Neural Differentiation in the Stem Cell of the Human Periodontal Ligament

**DOI:** 10.1038/s41598-018-27492-0

**Published:** 2018-06-14

**Authors:** Letizia Romeo, Francesca Diomede, Agnese Gugliandolo, Domenico Scionti, Fabrizio Lo Giudice, Veronica Lanza Cariccio, Renato Iori, Placido Bramanti, Oriana Trubiani, Emanuela Mazzon

**Affiliations:** 1 IRCCS Centro Neurolesi “Bonino-Pulejo”, Via Provinciale Palermo, Contrada Casazza, 98124 Messina, Italy; 2 Department of medical, oral and biotechnological sciences, University “G. d’Annunzio” Chieti-Pescara, Chieti, Italy; 3 Consiglio per la ricerca in agricoltura e l’analisi dell’economia agraria, Centro di ricerca Agricoltura e Ambiente (CREA-AA), Via di Corticella 133, 40128 Bologna, Italy

## Abstract

The therapeutic strategies for neurodegenerative diseases still represent a vast research field because of the lack of targeted, effective and resolutive treatment for neurodegenerative diseases. The use of stem cell-based therapy is an alternative approach that could lead to the replacement of damaged neuronal tissue. For this purpose, adult mesenchymal stem cells (MSC), including periodontal ligament stem cells (PDLSCs), could be very useful for their differentiation capacity, easy isolation and the ability to perform an autologous implant. The aim of this work was to test whether the Moringin [4-(α-L-rhamnosyloxy) benzyl isothiocyanate; GMG-ITC], an isothiocyanate extracted from *Moringa oleifera* seeds, was able to induce PDLSCs toward neural progenitor differentiation. Next-generation transcriptomics sequencing showed that moringin treatment increased the expression of genes involved in neuron cortical development and in particular in neuron belonging to upper and deep cortical layers. Moreover, moringin treatment upregulated genes involved in osteogenesis and adipogenesis although with a lower fold change compared to upregulated genes involved in neuronal differentiation. Finally, moringin did not induce the expression of oncogenes resulting in a safe treatment.

## Introduction

Neurological disorders, including Alzheimer’s disease, Multiple Sclerosis and Stroke result in the loss of neurons or progressive neuron dysfunction that lead to movement and balance impairment, vision defect and disturbance of consciousness^1^. Oxidative stress and inflammation play a pivotal role. Despite the worrying outcomes of these diseases, the high number of affected people and the outstanding health care costs, there is a substantial lack of treatment options. A novel therapeutic strategy is provided by the use of stem cell therapy^2^. A lot of evidences indicated that the differentiation ability of multipotent stem cell may be used as a source to replace lost neurons. Moreover, their neuronal protection/repair potential may allow to generate a micro-environment useful for neuronal development^3–7^. Mesenchymal stem cells (MSCs) are the most deeply studied adult stem cells to be potentially used for cell-therapy against neurological disorders^8^. Moreover MSCs are able to release growth factors, cytokines, and chemokines, playing a significant biological role during injury^9^. MSCs are present in adult bone marrow and are described as multipotent cells able to differentiate into mesenchymal derived tissues including bone, cartilage, fat, tendon, muscle, and neuronal tissues^10^. Oral cavity represents an important source of MSCs including human gingiva-derived mesenchymal stem cells (hGMSCs) and dental tissue derived MSCs such as dental pulp stem cells, stem cells from human exfoliated deciduous teeth, human periodontal ligament stem cells (hPDLSCs), stem cells from apical papilla and dental follicle progenitor cells. All together these cells originate from cranial neural crest and are prone to differentiate into neuronal cells^11–15^. It was reported that the prolonged expansion of stem cells may induce spontaneous differentiation into neuronal progenitor cells^16^.

Phytocompounds properties showed beneficial activities on human health. In particular, isothiocyanates were able to exert neuroprotective effects against neurodegenerative disease^17^. Among these, moringin [4-(α-L-rhamnosyloxy)-benzyl isothiocyanate], extracted from *Moringa Oleifera* seeds^18,19^ induced health promoting effects. Interestingly, moringin has shown to have not only an effective antitumor-promoting activity^20^ but also anti-inflammatory and antioxidant effects, protecting against neurodegenerative disorders^17,21,22^. Little is known about the potential role of *Moringa oleifera* treatment during cell differentiation. However, ethanol extract of *Moringa oleifera* leaf appears able to significantly promote the earlier stages of neuronal differentiation of mouse embryonic hippocampal neurons, increasing the number and length of dendrites, the length of axon and inducing synapses development^23^. To the contrary *Moringa oleifera* interfers with adipogenesis of human stem cells during the adipogenic differentiation determining a decrease of expression levels of the mRNA involved into the de novo synthesis of fatty acids (SREBP1c and FAS) and fatty acid uptake and transport (FABP4)^24^. In addition, ethanolic extract of *Moringa oleifera* leaf increases the osteogenic differentiation of porcine bone marrow derived MSCs, suggesting its potential use for bone regeneration^25^. Other phytochemicals coumpounds have been investigated for their properties to induce stem cell differentiation. Recently it has been discovered that low concentration of sulforaphane (SFN) is sufficient to stimulate rat embryonic neural stem cell proliferation and their differentiation to neurons increasing neurosphere formation^26^. In addition, Cannabidiol (CBD) from *Cannabis sativa* induces stem cell differentiation by significantly modulate the expression of genes involved in the early neuron differentiation, neurogenesis and nervous system development^27^.

In this study, we investigated whether moringin treatment may promote hPDLSCs differentiation toward a specific cortex neuronal developmental stage. Next Generation Sequencing (NGS) was performed on MiSeq Illumina instrument in order to analyze the change induced by three different doses of moringin on hPDLSCs transcriptomic profile with particular attention to the genes involved in neuronal differentiation. Moreover, the safety of moringin treatment and adipogenesis and osteoigenesis differentiation pathways were also investigated.

## Results

### Cytofluorimetric and morphological evaluation of hPDLSCs

**Figure 1:**
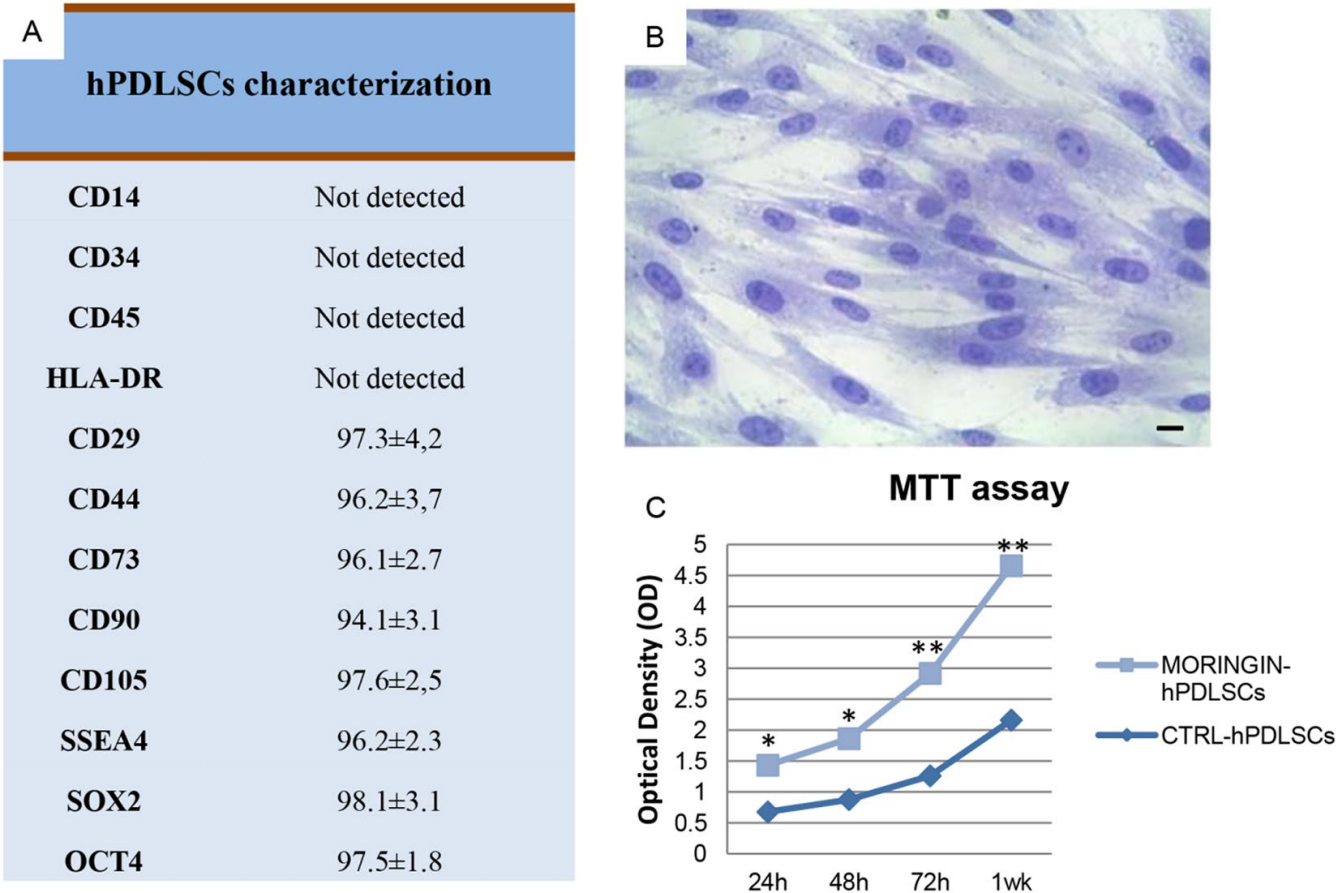
hPDLSCs characterization. (**A**) Cytofluorimetric evaluation of human PDLSCs surface markers. The numbers in the panel represented the percentage of markers expression in the cells (**B**) hPDLSCs stained with toluidine-blue solution. Scale bar: 10 μm (**C**) hPDLSCs were treated with moringin at a concentration of 0.5 μM for 24 h, 48 h, 72 h and 1 week of culture. The cell proliferation was determined using MTT assay. The means ± SEM of three independent experiments performed in triplicate are shown. *P < 0.05; **P < 0.01.

hPDLSCs were characterized for the expression of surface stem cell markers. Surface markers evaluation was carried out by flow cytometric analysis using three independent samples from three different patiens. hPDLSCs showed to be positive for CD29, CD44, CD73, CD90, CD105, SSEA4, SOX2 and OCT4, while all cells were negative for the following markers: CD14, CD34, CD45 and HLA-DR (Fig. 1A). Plastic adherent hPDLSCs, observed at inverted light microscopy, showed a fibroblastic like morphology with elongated cytoplasmic process, elliptic nuclei with one or two evident nucleoli (Fig. 1B). Moringin treatment did not change cell morphology (data not shown).

### Proliferation Analysis

MTT proliferation assay kit was used to evaluate hPDLSCs viability proliferations at 24 h, 48 h, 72 h, and 1 week of culture. Logarithmic viability rate was reported for both treated and untreated samples. Results indicated that moringin treatment did not induce cell death but on the contrary determined an increase of hPDLSCs proliferation rate compared to control sample. In particular, hPDLSCs treated with 0.5 μM of moringin showed statistically significant differences after 72 h and 1 week of culture when compared to the control cells (Fig. 1C).

### Transciptome analysis of CTR-hPDLSCs and MORINGIN-hPDLSCs

**Figure 2:**
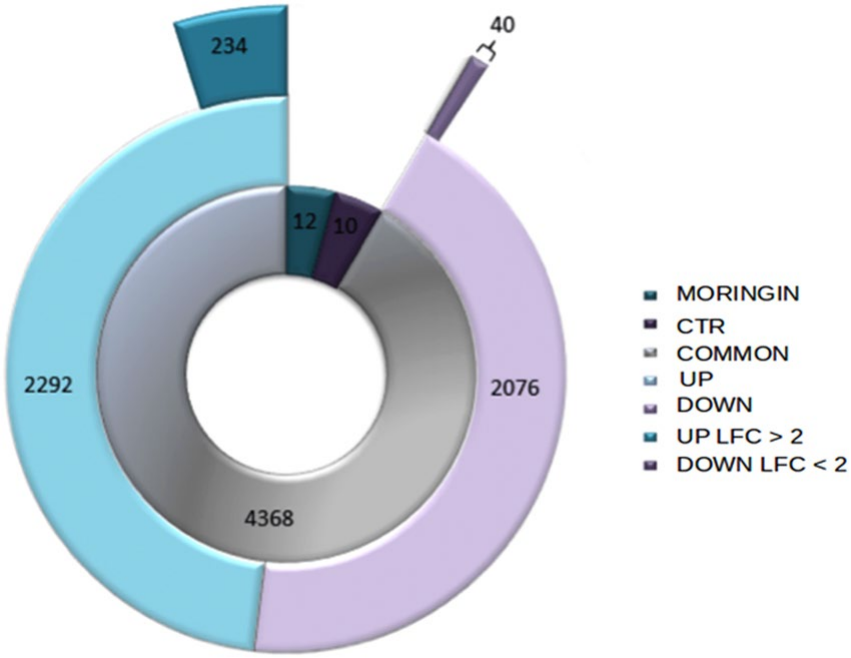
Global gene expression. In the chart, the inner ring corresponds to the total number of genes statistically significant, among these the gray part (4368) represents the common genes differentially expressed in both conditions, which are control (CTR-hPDLSCs) and cells treated with 0.5 μM of moringin (MORINGIN-hPDLSCs). Instead the two remaining small parts of the inner ring indicate the number of genes expressed only in the CTR-hPDLSCs (10) and in the MORINGIN-hPDLSCs (12). In the second ring the portion of common genes is divided into upregulated (2076) and downregulated (2292) genes. The third ring shows the genes upregulated (40) and downregulated (234) that have a Log2 Fold Change (LFC) > 2.

**Figure 3:**
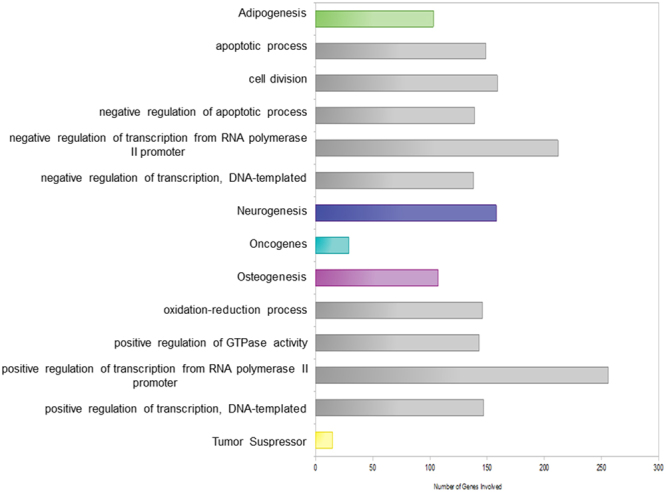
Categories of common differentially expressed genes in hPDLSCs treated with 0.5 μM of moringin. The bar chart shows in Y axis the genes differentially expressed grouped by the main categories obtained through public databases (GO, CORTECON, NCBI gene, ATLAS, TSGene and NCG5.0); X axis shows the number of genes involved in each gene category.

The transcriptome of hPDLSC treated with moringin (0.5 μM) (MORINGIN-hPDLSCs) and untreated cells (CTR-hPDLSCs) was investigated by NGS analysis. Trascriptome analysis was carried out using three independent samples from three different patients. Global gene expression has shown that 4390 genes were differentially expressed between CTR-hPDLSCs and MORINGIN-hPDLSCs, among which 12 genes were expressed only in MORINGIN-hPDLSCs and 10 only in CTR-hPDLSCs, while 4368 genes were common between the two groups (Fig. 2). MORINGIN-hPDLSCs and CTR-hPDLSCs exclusively expressed genes did not belong to a specific gene ontology and, thus, they were not included in a specific family. Of the common genes, 2292 were upregulated while 2076 were downregulated in MORINGIN-hPDLSCs, as shown in Fig. 2. All genes were analyzed by NCBI gene and classified by Gene Ontology (GO). Analysis using public databases (GO, CORTECON, NCBI gene, ATLAS, TSGene and NCG5.0) lead us to identify genes belonging to general family (in grey color in the Fig. 3) and genes involved in neurogenesis (identified using CORTECON database), adipogenesis and osteogenesis. Moreover, in order to evaluate the safety of the moringin treatment, oncogenes and tumour suppressor were identified by ATLAS, TSGene and NCG5.0 database and analyzed for their expression level (Fig. 3).

### Differentially expressed genes involved in neurogenesis and neural differentiation

**Figure 4:**
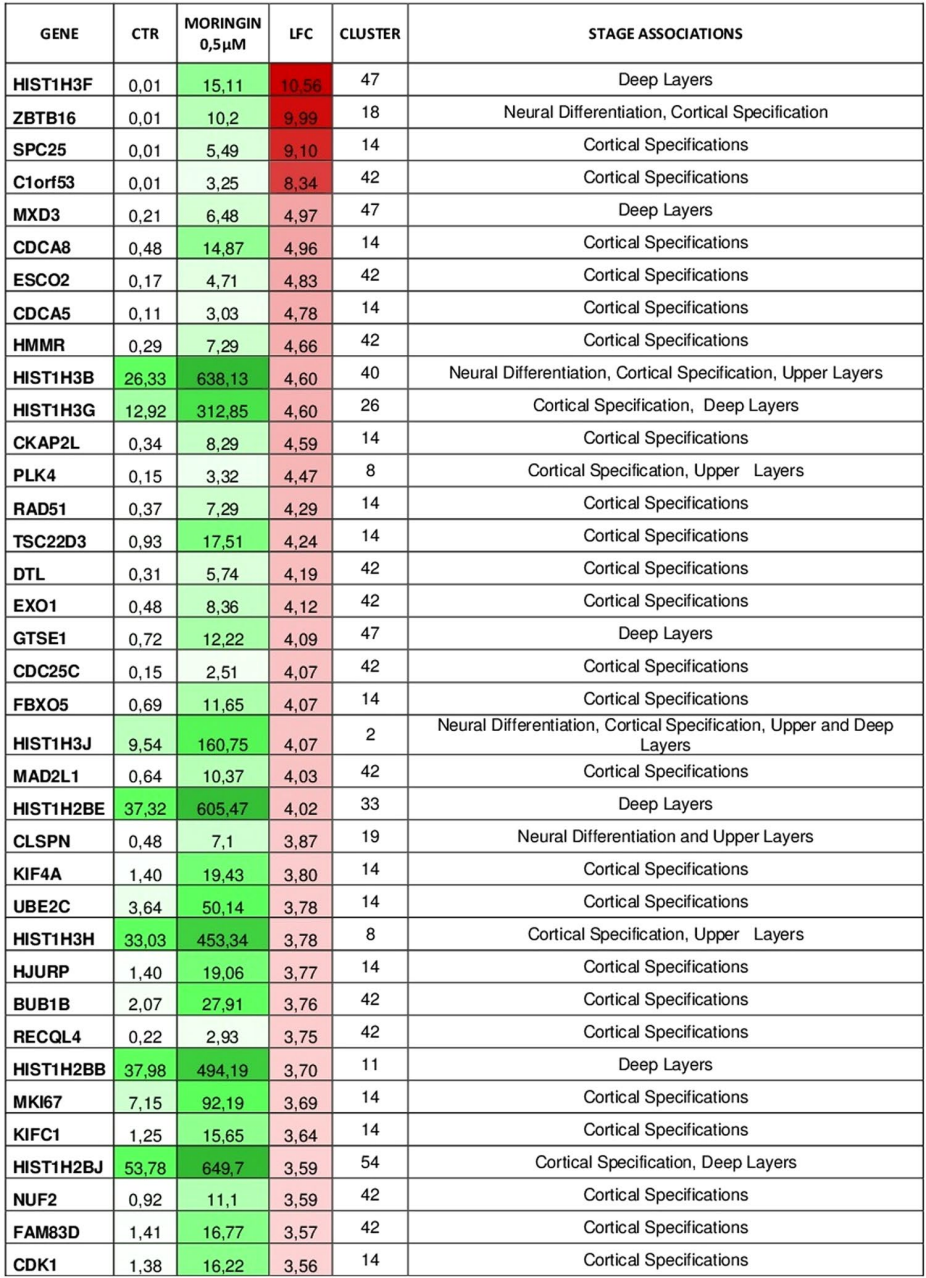
Differentially expressed genes involved in cerebral cortex development induced by 0.5 μM of moringin. Heat maps show differentially expressed genes that have Log2FoldChange (LFC) > 2 (red scale) and a q-value (FDR) < 0.05, hence genes that after treatment with 0.5 μM of moringin appear overexpressed and statistically significant. The expression’s values in the two conditions, CTR-hPDLSCs and MORINGIN-hPDLSCs, are indicated through the FPKM (Fragments Per Kilobase of exon model per Million mapped fragments) and graphically depicted in green scale, where a different intensity of color represents a differential gene expression, that is also reported in column 4 as fold change value (red color). In column 4 a more intense red color is associated to a higher fold change value. Gene cluster and stage association analyzed by Cortecon database were also shown.

Among 2292 genes that were up regulated in 0.5 μM MORINGIN-hPDLSCs compared to CTR-hPDLSCs, 234 genes showed a differential gene expression level with fold change >2 and q-value < 0.05 after moringin treatment. Using CORTECON database we have found that among 234 genes with fold change > 2, 158 were genes involved in cerebral cortex development (Fig. 4 and Tables S1–S4 and S5–S9). Cortecon analysis of the 158 genes up regulated in 0.5 μM MORINGIN-hPDLSCs indicated that 43 genes were included in the gene cluster 42 anld 54 genes were included in the gene cluster 14. Both 42 and 14 gene clusters encompassed genes associated to cortical developmental stage indicated as cortical specification (CS) (Fig. 4 and Tables S1–S4 and S5–S9). The remaining 61 genes belonged to gene clusters associated to different categories of developmental and differentiation stages indicated by Cortecon database as Neural Differentiation (ND), Cortical Specification (CS), Upper Layer (UL) and Deep Layer (DL). These results showed that 0.5 μM moringin treatment not only improve hPDLSCs differentiation toward the neuronal lineage but induced cellular reprogramming to different neuronal cortex development stage as indicated in Fig. 4 and Tables S1–S4.

**Figure 5:**
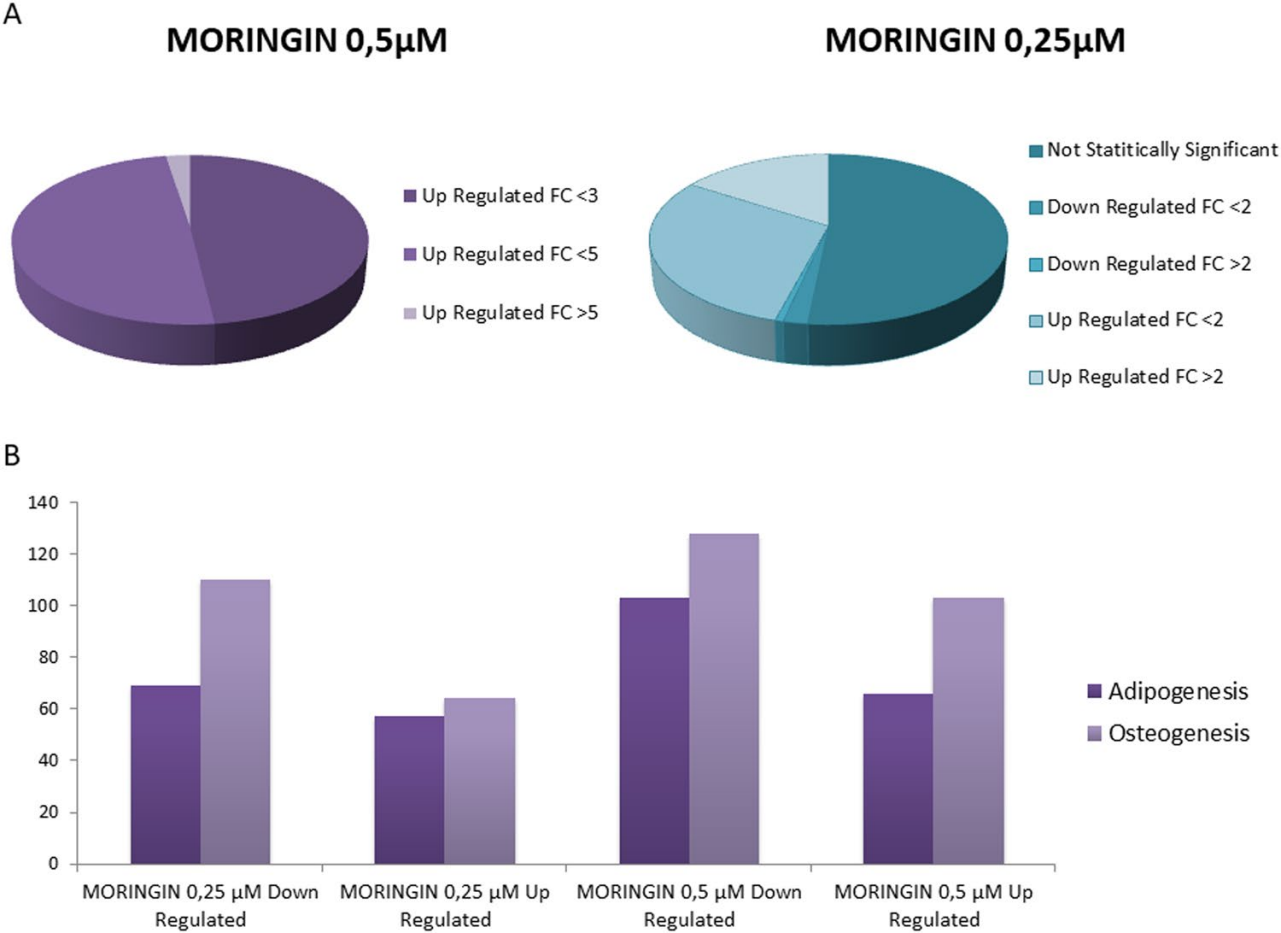
(**A**) Differentially expressed genes involved in neurogenesis in hPDLSCs treated with 0.5 μM and 0.25 μM of moringin. Genes involved in neurogenesis analyzed in hPDLSCs treated with 0.5 μM and 0.25 μM of moringin by CORTECON database were divided for their fold change (FC). (**B**)Upregulated and downregulated genes involved in osteogenesis and adipogenesis in hPDLSCs treated with 0.5 μM and 0.25 μM of moringin. Upregulated and downregulated genes involved in osteogenesis and adipogenesis were compared in hPDLSCs treated with 0.5 μM and in hPDLSCs treated with 0.25 μM.

Moreover, we also evaluate the effect of different doses of moringin on the hPDLSCs neurogenic gene expression level. Results indicated that a treatment with a lower concentration of moringin, namely 0.25 μM, caused a dose dependent response (Tables S5–S9). Indeed looking at 158 genes upregulated by 0.5 μM of moringin, some of these were upregulated also with 0.25 μM of moringin but the fold change was lower compared to the higher dose. Particularly, the treatment with 0.25 μM of moringin determined the up regulation of only 73 genes but only 24 genes showed a fold change >2. To the contrary 3 genes were also down regulated, while 81 were not significantly changed compared to the control (Fig. 5A). Finally a dose of 1 μM of moringin induced the same results (data not shown) obtained using 0.5 μM of moringin, the higher dose indicated in the experimental results.

### Differentially expressed genes involved in osteogenesis/adipogenesis

By comparison with Bionaz *et al*. research (2015)^28^, we have found among our data a significant modulation of 107 genes involved in osteogenesis (Tables S10–S11) and 103 genes involved in adipogenesis after 0.5 μM of moringin treatment (Tables S12–S13). Among osteogenesis involved genes, 50 were up regulated after moringin treatment and 57 down regulated. Moreover, only 39 genes were up regulated in adipogenesis instead 64 were down regulated. Only four genes (MYBL2, E2F1, BRCA1 and F3 for osteogenesysis and TSC22D3, KIAA1524, DKK1 and SLC29A1 for adipogenesyis) showed fold change >2 in both differentiation pathways, compared to the 158 genes involved in neuronal differentiation. To validate our results, we analyzed all differential-expressed genes by GO in order to find those involved in osteogenesis and adipogenesis processes, as “ossification”, “osteoblast differentiation”, “regulation of ossification”, “fat cell differentiation” and “regulation of fat cell differentiation”. For osteogenesis we found 59 up regulated genes (Table S14) and 82 down regulated ones (Tables S15–S16). In adipogenesis process 35 up regulated genes were found, while 39 genes were down regulated (Table S17). We also assessed wheter similar results would be obtained using a different dose of moringin (0.25 μM). Our results indicated that the genes involved in osteogenesis and adipogenesis processes were regulated in a dose dependent manner (Fig. 5B). Indeed, only 64 genes involved in osteogenesis were upregulated by 0.25 μM of moringin compared to the 103 genes that were induced by a treatment with 0.5 μM of moringin. In accordance, 0.25 μM of moringin determined the downregulation of 110 genes while 128 were downregulated by 0.5 μM of moringin. Concerning adipogenesis, 57 genes were upregulated after a treatment with 0.25 μM of moringinwhile 66 were upregulated by 0.5 μM of moringin. Moreover, 69 genes were dowregulated by 0.25 μM of moringin compared to the 103 genes downregulated by a treatment with 0.5 μM of moringin.

### Osteogenic and adipogenic induction

**Figure 6:**
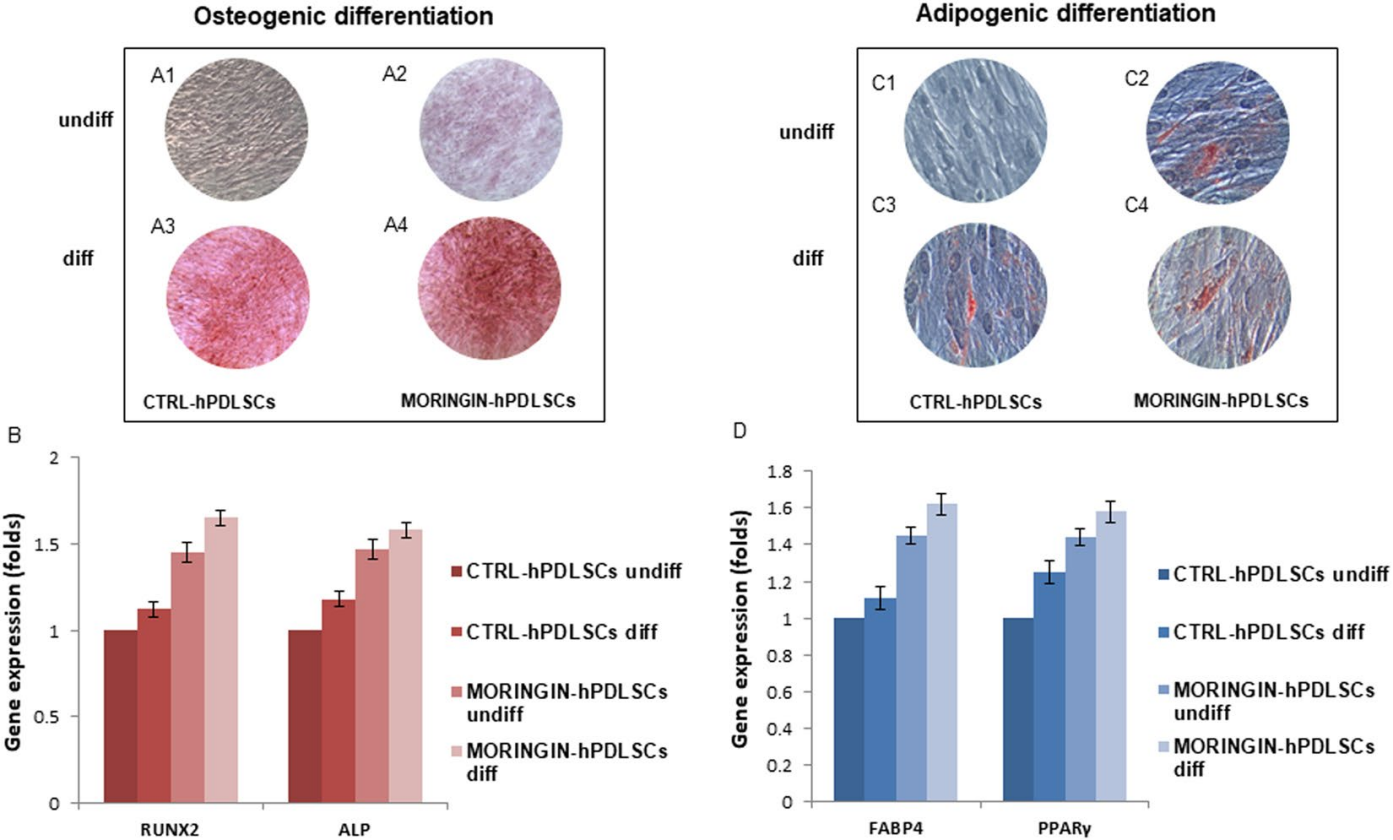
hPDLSCs mesengenic differentiation. Images of alizarin red s staining in CTRL-hPDLSCs and 0.5 μM MORINGIN-hPDLSCs after 15 days of culture in undifferentiated (**A1**-**A2**) and differentiated conditions (**A3**-**A4**). RT-PCR bars graph of osteogenic markers (**B**). Images of Adipo oil red staining in CTRL-hPDLSCs and 0.5 μM MORINGIN-hPDLSCs after 15 days of culture in undifferentiated (**C1**-**C2**) and differentiated conditions (**C3**-**C4**). RT-PCR bars graph of adipogenic markers (**D**). A1-A4 Bar: 100 µm. (**C1**-**C4**): Bar: 50 µm.

Osteogenic differentiation was evaluated after 15 days with alizarin red solution staining. The precence of calcium precipitates was evidenced in differentiated CTRL-hPDLSCs and 0.5 μM MORINGIN-hPDLSCs and also in undifferentiated MORINGIN-hPDLSCs, but at a lower level (Fig. 6A1–A4). RUNX-2 and ALP genes were analyzed to validate the osteogenic commitment, in fact an increased osteogenic gene expression was present in 0.5 μM MORINGIN-hPDLSCs (Fig. 6B), especially in those under osteogenic differentiation condition. Adipogenic differentiation was analysed by means adipo oil red staining. Red-lipid vacuoles at cytoplasmic level indicated an early adipogenic commitment in undifferentiated 0.5 μM MORINGIN-hPDLSCs. A positive staining was visible in differentiated samples (Fig. 6 C1–C4). The upmodulation of FABP4 and PPARɣ genes validate the adipogenic induction process (Fig. 6D).

### Neurogenic induction

**Figure 7:**
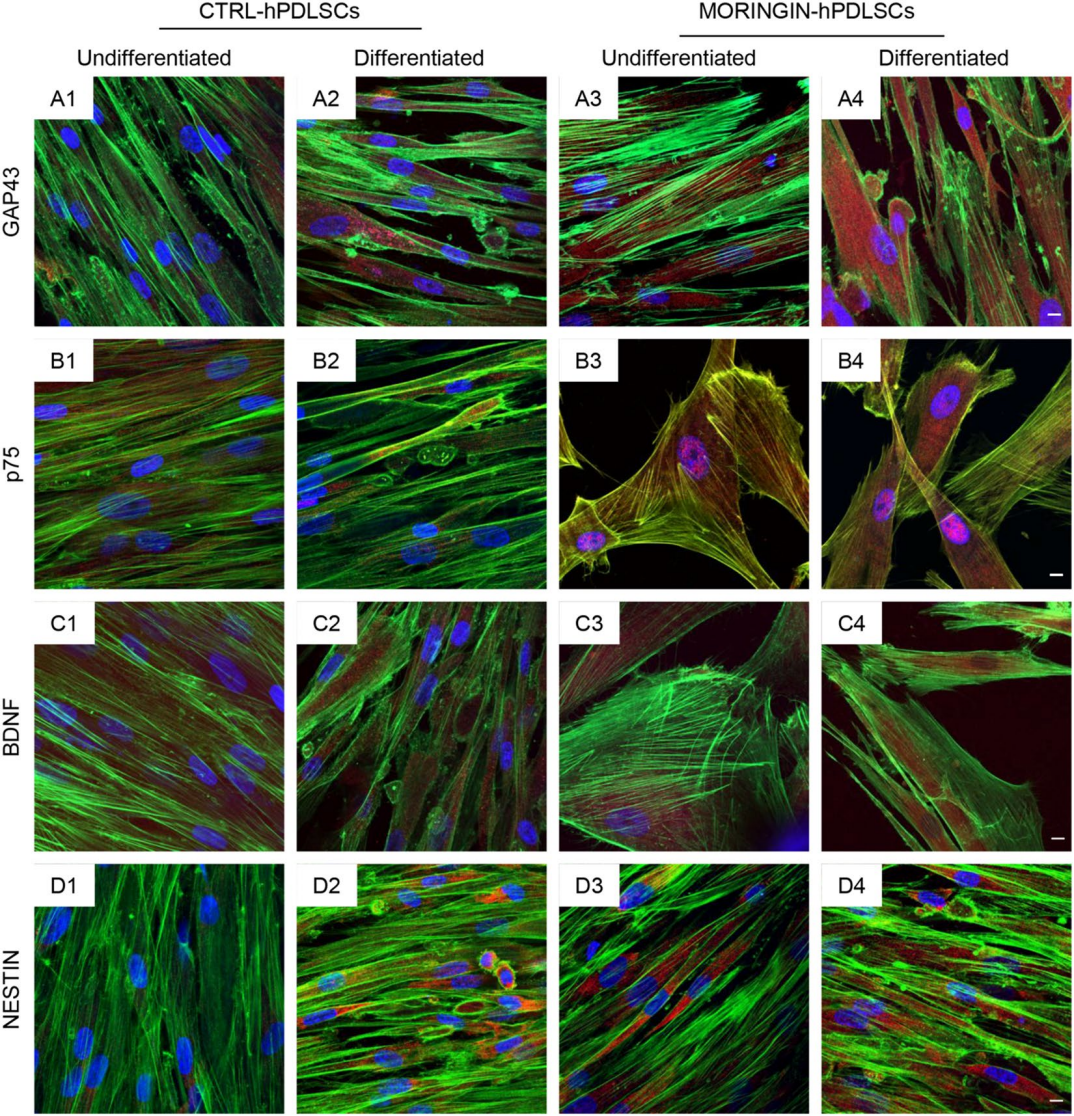
hPDLSCs neurogenic differentiation. Images of immunofluorescence staining in CTRL-hPDLSCs and MORINGIN-hPDLSCs after 10 days of culture in undifferentiated and differentiated conditions. Evaluation of different expression level of GAP43 (**A1**,**A2**,**A3**,**A4**), p75 (**B1**,**B2**,**B3**,**B4**), BDNF (**C1**,**C2**,**C3**,**C4**) and NESTIN (**D1**,**D2**,**D3**,**D4**). Mag: 40×. Bar: 50 µm.

After 10 days of culture in neuronal induction medium differentiated CTRL-hPDLSCs ad MORINGIN-hPDLSCs were observed at confocal laser scanning microscopy. GAP43, p75, BDNF and NESTIN were up-regulated particularly in MORINGIN-hPDLSCs when compared to the untreated cells **(**Fig. 7). P75 is translocated at nuclear level in MORINGIN-hPDLSCs.

### Oncogenes expression

In order to evaluate the safety profile of 0.5 μM moringin treatment on hPDLSCs we analyzed the differential expression level of oncogenes and tumour suppressor induced by moringin treatment in MORINGIN-hPDLSCs compared to CTR-hPDLSCs (Table S18). Oncogenes and tumour suppressor were identified by ATLAS, TSGene and NCG 5.0 database and analyzed for their expression level. 29 oncogenes were identified and, among these, 8 (BCL2, CDX2, EVI1, LCK, MYB, MYCN, TCL1A, USP6) were not expressed in both condition MORINGIN-hPDLSCs and CTR-hPDLSCs.

The upregulated genes (BCL11A, ETV4, CCND2, MAFB, PIM1, MAML2, CBLB) showed increased fold change, although this increment was not statistically significant. Among the other genes only 5 genes (AKT2, CCND1, ERBB2, MAF, SMO) were statistically supported but moringin treatment induced their downregulation. A total of 15 tumour suppressor genes were identified. IL2 and SYK were not expressed in moringin treated and untreated cells. The majority of genes were downregulated but showed q-value > 0,05 and thus resulted statistically irrelevant. Only one gene, PALB2, showed q-value < 0,05 but was upregulated after moringin treatment.

## Discussion

Stem-cell therapy represents an alternative strategy against neurological damage that occurs in many neurodegenerative diseases, as Alzheimer’s disease, Multiple Sclerosis and Stroke. MSCs including hPDLSCs were the most promising source to replace lost neurons by their multipotent differentiation ability and their natural propensity to differentiate into neuronal cells. In this study, we investigated whether the use of a phyto-compound could be able to address hPDLSCs differentiation toward a specific neuronal cell type. For this purpose, we analyzed the hPDLSCs transcriptome profile induced by moringin (4-(α-L-rhamnosyloxy) benzyl isothiocyanate) ITC extract isolated from *Moringa oleifera* seeds that we have already deeply investigated for its neuroprotective effects^17,21,22^. Before treatment we carried out cytofluorimetric analysis on isolated hPDLSCs to analyze the superficial markers of stem cell. As expected, staminal phenotype of hPDLSCs was characterized by expression of MSC surface markers (CD105, CD90, CD73, CD29, CD44, SSEA4, SOX2 and OCT4) while CD45, CD34, CD14 and HLA-DR were not present^29^.

Moreover, hPDLSCs showed a fibroblastic like morphology, that did not change after the treatment with moringin. After the treatment, we also assessed its effects on hPDLSCs cell proliferation by MTT assay. Our results showed that 0.5 μM moringin treatment induced a significant time-dependent increase of viability rate compared to untreated cells, indicating that moringin treatment did not impair hPDLSCs survival but to the contrary improve cell proliferation. Then we carried out NGS analysis to investigate about the differential hPDLSCs transcriptional profile induced by moringin treatment in MORINGIN-hPDLSCs in comparison with CTR-hPDLSCs (untreated cells). hPDLSCs were treated with 0.25 μM, 0.5 μM, and 1 μM of moringin. NGS analysis showed that the lower dose of moringin induced, in a dose dependent manner, the expression of genes involved in neuronal differentiation. On the contrary, to the upper dose of 0.5 µM (1 μM) the fold change was not dose-dependent. On the bases of these results, we carried out our experiments using 0.5 μM of moringin.

NGS analysis highlighted a total of 4368 common genes differentially expressed between 0.5 μM moringin treated and untreated cells. Among these, moringin treatment induced the downregulation of 2076 and the upregulation of 2292 genes. Interestingly, up regulated genes included a great number of genes (234) with fold change >2. Since hPDLSCs are able to differentiate into different cell line including neuronal, osteoblastic and adipocyte cells, we first investigated about hPDLSCs neuronal differentiation induced by moringin treatment as hypothetical strategy for a successful stem cell therapy against brain injury. Analysis of up regulated genes with fold change >2 was done by Cortecon database. As described Cortecon is a database that provides online, query-based access to changes in RNA expression and alternatively spliced transcripts during human cortical development^30^. Based on their temporal expression profile during cortical development, genes were grouped into 64 clusters and organized into 5 different categories of developmental stage: Pluripotency (PP), Neural Differentiation (ND), Cortical Specification (CS), Deep Layer Neuron Generation (DL) and Upper layer Neuron Generation (UL)^30^. In addition, Cortecon examined each cluster using GO, KEGG pathway and diseases association^30^. Through Cortecon analysis, 158 genes with fold change >2 were associated with different stages of cortical development. Cortecon analysis lead us to identify that the majority of genes were included into cluster 42 (43 genes) and 14 (54 genes), associated with Cortical Specification development stage. Moreover, the remaining genes belonged to different clusters correlated with various stages of cortical development, including cortex deep layer and upper layer. These results could suggest that moringin treatment induce not only hPDLSCs neuronal differentiation but also reprogramming of cell differentiation toward cortex specific area. Moreover, it is important to note that moringin treatment within 48 h was sufficient to obtain hPDLSCs neuronal cortex differentiation. This result is surprising if compared with a previous study of our research group, where hGMSCs spontaneously differentiated toward neuronal cell line after the prolonged cellular expansion^16^. Indeed, neuronal progenitor markers and de novo expression of neural precursor genes were observed only in hGMSCs cultured until passage 41 than passage 10^16^. To the contrary moringin treatment induced early up regulation of neural development associated genes in hPDLSCs already within 48 h of treatment. In addition, a lower dose of moringin was less effective to induce the neuronal differentiation showing a dose dependent response. However, a higher dose of moringin (1 μM) did not exert increased effects on neuronal differentiation.

In order to assess if neuronal cell type was the main differentiation pathway preferentially induced by moringin treatment, osteogenesis and adipogenesis associated genes were also investigated. To evaluate osteogenesis and adipogenesis associated genes we used GO database and the comparison with the transcriptomic analysis performed by M. Bionaz et collaborators^28^. Furthermore, after 0.5 μM moringin treatment, the majority of differentially expressed genes involved in adipogenesis and osteogenesis showed a fold change <2, when compared with the regulated genes involved in the neurogenic pathway. In addition, only a few genes showed fold change >2 in both differentiation pathways, compared to the 158 genes involved in neuronal differentiation. Through GO analysis, we found that genes involved in osteogenesis belonged to the main GO categories, as “ossification”, “osteoblast differentiation” and “regulation of ossification” and for adipogenesis, genes were associated to the category: “fat cell differentiation” and “regulation of fat cell differentiation”. These findings are in accordance with Barbagallo *et al*., that suggested that *Moringa oleifera* did not induce adipogenic differentiation of human adipose stem cells in the presence of adipogenic differentiation stimuli^24^. To the contrary *Moringa oleifera* appeared to be able to induce neural differentiation of rat hippocampal neurons primary culture towards mature neuron^23^. Also for osteogenic and adipogenic differentiation a dose dependent response was observed and 0.5 μM of moringin dose was the most effective. Althought NGS analysis showed a lower expression level of genes involved in ostegenic and adipogenic processes, 0.5 μM of moringin determined in hPDLSCs cultured in osteogenic and adipogenic condition for 15 days a light increased expression of osteogenic and adipogenic markers.

Our results suggested that 0.5 μM of moringin treatment may be able to accelerate the differentiation process after a short time (48 h) and the main differentiation lineage induced was the neuronal one. Neuronal differentiation process, evaluated by means immunofluorescence expression of neuronal related markers, showed after 10 days of inductive culture, an increase of GAP43, p75, BDNF and NESTIN. In particular, our experiments evidenced that moringin treatment of hPDLSCs, not only determined an increase of P75 expression but also this upregulation is associated to its nuclear translocation. The ability of p75 to translocate at the nuclear region, in response to moringin treatment, is particularly intriguing because probably it acts as ligand starting a neuronal differentiation pathway. The p75 neurotrophin receptor is the first member of the tumor necrosis factor receptor superfamily that includes the Fas antigen, DR6, CD30, and CD40^31^. The p75 receptor is also recognized by all the neurotrophins (NGF,3 brain-derived neurotrophic factor, neurotrophin-3, and neurotrophin-4), and it is able to promote differentiation, growth, and survival of different cell types in the nervous system^32^. Finally, analysis of differential expression oncogenes and tumour suppressor was carried out to verify if moringin treatment could address hPDLSCs toward tumorigenic process. Our results indicated that moringin treatment did not induce a significant change for oncogenes expression and tumor suppressor genes.

## Conclusions

All together, our results indicated that moringin treatment is a promising strategy to promote and accelerate hPDLSCs cortex neuronal differentiation in order to improve stem-cell therapy against neurological disorders. Moreover, moringin treatment seems to be safe given that tumorgenic processes are not activated.

## Materials and Methods

### Purification of moringin

Moringin was isolated from *M. oleifera* (fam. Moringaceae) seeds (cake powder PKM2 provided by Indena India Pvt. Ltd.; Bangalore, India) at the Bologna laboratory (CREA-AA; previously CIN) using established methods and the structure was confirmed by nuclear magnetic resonance (NMR) spectroscopic analyses^18,19^.

### Ethic Statement

The protocol for cell isolation and culture, was approved by the Ethical Committee at the Medical School, “G. d’Annunzio” University, Chieti, Italy (number 266/April 17, 2014). All patients enrolled in the study signed the informative consent form before tissue collection and all experiments were performed in accordance with relevant guidelines and regulations.

### Cell Culture

Cells derived from periodontal tissue were collected as previously described, scraping a third coronal root surface using Gracey’s curette^33^. All three patients, included in the study, were in good general health and exempt from oral and systemic diseases. After collection, hPDLSCs were cultured using MSCGM-CD medium (mesenchymal stem cell growth medium chemically defined) (Lonza, Basel, Switzerland) and were maintained in an incubator at 37 °C in a humidified atmosphere of 5% CO2 in air. To evaluate cells morphological features, plastic-adherent hPDLSCs were stained used toluidine blue solution and then observed at inverted light microscopy DMIL (Leica Microsystem, Milan, Italy)^34^.

### Cytofluorimetric Evaluation

The cytofluorimetric evaluation of the surface markers was carried out on hPDLSCs derived from three different patients and each hPDLSCs was assessed in triplicate. Samples were stained for surface or intracellular antigens (Table 1), as previously described by Rajan *et al*.^35^. Data were analyzed using a FACStar-plus flow cytometry system and the FlowJo™ software (TreeStar, Ashland, OR, USA).

**Table 1:** List of antibodies against surface or intracellular antigens used for cell characterization by cytofluorimetric analysis.

Antibody	Manufacturer
CD44-FITC	Ancell (MN, USA)
CD45-FITC	
CD29-PE	
CD105-FITC	
CD14-FITC	Miltenyi Biotec (Bergisch Gladbach, Germany);
HLA-DR-PE	Becton Dickinson (San Jose, CA, USA)
CD90-FITC	
CD73-PE	
SOX2-Alexa488	
SSEA4-FITC	
OCT3/4-PE	
CD34-PE	Beckman Coulter (Fullerton, CA, USA)
Fluorescein isothiocyanate (FITC); Phycoerythrin (PE).	

### Moringin treatment

In order to assess the possible cytotoxic effect of moringin on hPDLSCs, cells at 2^nd^ passage, grown until up 80% confluence were incubated with moringin at a concentration of 0.5 μM, (dissolved in 0.1% DMSO) for 24, 48, 72 h and 1 week. To perform transcriptomic analysis, hPDLSCs were treated with 0.5 μM and 0.25 μM of moringin (dissolved in 0.1% DMSO) for 48 h. The evaluation of the cytotoxic effect of moringin on hPDLSCs and the transcriptomic analysis were carried out using hPDLSCs derived from three different patients obataining three different groups. Each group was treated with moringin and 0.1% of DMSO that represented the control. Experiments were done by assessing the same sample in triplicate.

### Cell Proliferation Assay

The proliferation ability of hPDLSCs cultured with or without moringin at a concentration of 0.5 μM, for 24, 48, 72 h and 1 week, were determined using the 3-(4,5-dimethylthiazolyl-2)-2,5-diphenyltetrazoliumbromide (MTT) method. 2000 cells/well were placed in a 96-well tissue culture plates and incubated at 37 °C. At designed time point, MTT solution (20 μl) (Promega, Milan, Italy) was added to each well to detect the metabolic activity of the cells. Indeed, living cells are able to reduce yellow tetrazole to purple insoluble formazan. A solubilization solution is than used to dissolve the insoluble purple formazan products into a colored solution. The absorbance level of MTT cleavage was directly proportional to the number of viable cells and indirectly indicate the proliferation rate after moringin treatment. All plates were cultured in the dark for 3 h at 37 °C^36^. Supernatants were read at 650 nm wavelength using a microplate reader (Synergy HT, BioTek Instruments, Winooski, VT, USA).

### Total RNA extraction and Illumina cDNA library construction

Total RNA was extracted from alls samples by using Reliaprep RNA Cell Miniprep System (Promega, USA). Every patient derived sample group was used as a control (sample treated only with 0.1% of DMSO) and as a test (sample treated with moringin resuspended in PBS with 0.1% of DMSO) to have sample matched. Experiments were done by assessing the same sample in triplicate.

According to the TruSeq RNA Access library kit protocol (Illumina, San Diego, CA), 40 ng of total RNA has been fragmented by setting a thermal cycler to 94 °C for 8 minutes, obtaining fragments >200 nt, that have been used for first strand cDNA synthesis by SuperScript II reverse transcriptase activity (Invitrogen, Carlsbad, CA). A double strand cDNA has been synthesized by incubation at 16 °C for 1 hour with Second Strand Marking Master Mix and then purified by AMPure XP beads in order to eliminate the reaction mix. By fragments adhenilation at 3’ ends, the ligation of complementary adapters (showing a “T” nucleotide to the 3′ ends) is possible together with a reduced rate of chimera (concatenated template) formation. The next step of Adapter-Indexes ligation to ds cDNA fragment allows the identification of each sample and prepares them for hybridization on flow cell. After a clean-up step, a first PCR amplification was performed according to the following program: initial denaturation at 98 °C for 30 s, 15 cycles consisting of 98 °C for 10 s, 60 °C for 30 s, 72 °C for 30 s and final extension at 72 °C for 5 minutes. A first hybridization reaction permits to mix cDNA library with exome capture probes in order to select and to enrich particular regions of interest. Hybridization reaction (95 °C per 10 min, 18 cycles of 1 min incubation, starting at 94 °C, decreasing 2 °C per cycle and 58 °C for 90 min.) allows to obtain a pool of different indexing libraries, by using 200 ng of each one. A streptavidin conjugated magnetic beads are used to purify sample pool and a second hybridization step followed by another streptavidin purification have been performed before a final PCR amplification (98 °C for 30 s, 10 cycles: 98 °C for 10 s, 60 °C for 30 s, and 72 °C for 30 s, and 72 °C for 5 min.) and successive clean-up. Finally, the library has been qualitatively and quantitatively validated by Bioanalyzer instrument (Agilent High Sensitivity DNA Kit, Richardson, TX) and Real-Time PCR (KAPA Library Quantification Kit-Illumina/ABI Prism® (Kapa Biosystems, Inc., Wilmington, MA), respectively, and had been denatured by 2 N NaOH and diluted until 12 pM concentration. MiSeq Reagent Kit v3 (150 cycles) has been used for sequencing on the MiSeq Instrument (Illumina), by setting a single read.

### MiSeq-generated data processing and Statistical analysis

The CASAVA software version 1.8 (Illumina) was used for generated the “Fastaq.File”. The file.fastaq are aligned using the tool STAR, the “*homo sapiens* UCSC hg19” reference sequence was used for mapping. To evaluate the rate of differentially expressed genes between the different experimental groups that were statistically relevant, we carried out the statistical analysis with the Cufflinks Assembly & DE package version 2.0.0.

In order to normalize and compare all samples, the FPKM value (fragments per kilobase of exon per million fragments mapped) was calculated by applying the mathematical formula: (1000 × read count)/(number of gene covered bases × number of mapped fragments in million). A scatter plot of the Log_2_ of the FPKM was used in order to compare the two experimental groups. NCBI website (http://www.ncbi.nlm.nih.gov/gene), Gene Ontology (http://www.geneontology.org/), Cortecon (http://cortecon.neuralsci.org/), Atlas (https://www.proteinatlas.org/), TSGene (https://bioinfo.uth.edu/TSGene/) and NCG 5.0 (http://ncg.kcl.ac.uk/) they have been used to investigate and classify the differentially expressed genes between the two sperimental groups.

### Osteogenic and adipogenic differentiation

hPDLSCs treated or not with moringin 0.5 μM were seeded at a density of 5 × 10^4^ cells/cm^2^. Cells were maintained under standard and adipogenic or osteogenic conditions for 15 days. The medium was removed and replaced every three days. After differentiation period, the formation of mineralized precipitates or lipid vacuoles were assessed by alizarin red and adipo oil red staining as previously described by Libro *et al*.^37^ and observed at inverted light microscopy Leica DMIL (Leica Microsystem). To validate osteogenic and adipogenic differentiation RUNX-2, ALP, FABP4 and PPARγ were evaluated as reported by Ballerini *et al*.^38^. Each sample was run as triplicate. The ΔΔCt method was used to compare relative fold changes between samples and controls. T-test was used to assess the p-value, considering data significant when p < 0.05.

### Neuronal differentiation

CTRL and MORINGIN-hPDLSCs were cultured in undifferentiated and neurogenic differentiated medium for 10 days. Differentiation medium was composed by Neurobasal-A Medium(Gibco®) containing B27 (2%), L-glutamine (2 mM), penicillin (100 U/ml), streptomycin (100 mg/ml) and amphotericin B (5 mg/ml) and supplemented with basic Fibroblast Growth Factor (bFGF, 20 ng/ml) (TemaRicerca, Milan, Italy)^39^. Fixed cells were incubated with rabbit primary monoclonal antibody, anti-GAP-43 (rabbit, 1:200; Sigma Aldrich, Milan, Italy), anti-p75 (rabbit, 1:200; DBA, Milan, Italy), anti-BDNF (rabbit; 1:50; Santa Cruz Biotechnology, Inc., Dallas, TX, USA) and anti-Nestin (rabbit, 1:200; Santa Cruz Biotechnology)^40,41^. Cells were incubated with anti-rabbit Alexa Fluor 568 (Molecular Probes, Life Technologies, Monza, MI, Italy). All samples were incubated with Alexa Fluor 488 phalloidin green fluorescence conjugate (1:400), to mark the cytoskeleton actin and with TOPRO to staining nuclei. Confocal laser scanning microscopy (Zeiss LSM800, Zeiss, Jena, Germany), equipped with a Plan Neofluar oil-immersion objective (40×/1.3 NA) was used to samples observation.

### Data availability

Data are available from the corresponding author on reasonable request.

## Electronic supplementary material

Supplementary Dataset 1
