# Supplementary material for: Moringin Induces Neural Differentiation in the Stem Cell of the Human Periodontal Ligament
Source: Sci Rep. 2018 Jun 14;8:9153. doi: 10.1038/s41598-018-27492-0 (PMC6002387; doi:10.1038/s41598-018-27492-0)
Supplement: Supplementary file 1 — Supplementary Dataset 1 [file 41598_2018_27492_MOESM1_ESM.pdf]

MORINGIN INDUCES NEURAL DIFFERENTIATION IN THE STEM CELL OF THE HUMAN PERIODONTAL LIGAMENT

Letizia Romeo [1], Francesca Diomede [2], Agnese Gugliandolo[1], Domenico Scionti[1], Fabrizio Lo Giudice[1], Veronica Lanza Cariccio[1], Renato Iori [3], Placido Bramanti[1], Oriana Trubiani[2] and Emanuela Mazzon[1]*

1. IRCCS Centro Neurolesi "Bonino-Pulejo", Via Provinciale Palermo, Contrada Casazza, 98124 Messina, Italy;

2. Department of medical, oral and biotechnological sciences, University "G. d'Annunzio" Chieti-Pescara, Chieti, Italy;

3. Consiglio per la ricerca in agricoltura e l'analisi dell'economia agraria, Centro di ricerca Agricoltura e Ambiente (CREA-AA), Via di Corticella 133, 40128, Bologna, Italy.; renato.iori48@gmail.com

***Correspondence: emazzon.irccs@gmail.com; Tel.: +39-090-60128172**

GENE	CTR	MORINGIN 0,5µM	LFC	CLUSTER	STAGE ASSOCIATIONS
**TACC3**	2,08	24,51	3,56	1	Cortical Specification, Upper Layers
**RAD54L**	0,67	7,76	3,54	42	Cortical Specifications
**KIF18B**	0,26	2,95	3,53	42	Cortical Specifications
**AURKB**	0,51	5,93	3,53	14	Cortical Specifications
**TOP2A**	6,35	72,13	3,50	14	Cortical Specifications
**ESPL1**	0,24	2,74	3,49	42	Cortical Specifications
**KIF15**	0,34	3,79	3,49	41	Neural Differentiation
**CCNA2**	1,31	14,56	3,48	42	Cortical Specifications
**FAM72A**	0,31	3,51	3,48	14	Cortical Specifications
**GADD45B**	0,45	4,98	3,48	2	Neural Differentiation, Cortical Specification, Upper and Deep Layers
**ZWINT**	1,08	11,57	3,43	42	Cortical Specifications
**SGOL1**	0,24	2,58	3,43	14	Cortical Specifications
**BRIP1**	0,30	3,11	3,40	42	Cortical Specifications
**HIST1H2AG**	116,46	1201,16	3,37	2	Neural Differentiation, Cortical Specification, Upper and Deep Layers
**PSRC1**	0,36	3,74	3,36	14	Cortical Specifications
**DLGAP5**	1,97	20,05	3,35	14	Cortical Specifications
**BUB1**	1,04	10,47	3,33	42	Cortical Specifications
**KIF23**	1,74	17,52	3,33	42	Cortical Specifications
**ELN**	8,92	89,07	3,32	30	Cortical Specification, Upper and Deep Layers
**FANCA**	0,55	5,48	3,31	20	Deep Layers
**HIST1H2AE**	51,55	504,42	3,29	40	Neural Differentiation, Cortical Specification, Upper Layers
**ZNF724P**	1,44	14	3,28	14	Cortical Specifications
**CDC20**	3,38	32,36	3,26	14	Cortical Specifications
**SPC24**	2,32	22,22	3,26	14	Cortical Specifications
**NUSAP1**	2,45	23,29	3,25	37	Neural Differentiation
**FANCD2**	1,28	11,98	3,23	42	Cortical Specifications
**GAR1**	0,32	3,06	3,23	24	Cortical Specification, Upper and Deep Layers
**HIST1H4I**	12,55	114,71	3,19	62	Cortical Specification, Upper Layers
**RNASEH2A**	6,13	55,84	3,19	34	Neural Differentiation, Cortical Specification
**H2AFX**	6,02	54,91	3,19	14	Cortical Specifications
**HIST1H2BF**	100,56	917,01	3,19	11	Deep Layers
**KIF2C**	1,86	16,72	3,17	14	Cortical Specifications
**CASC5**	0,74	6,63	3,16	45	Cortical Specification, Upper and Deep Layers
**CEP55**	1,24	11,08	3,16	37	Neural Differentiation
**HIST1H3E**	2,03	17,9	3,14	42	Cortical Specifications

Table S1. Differentially expressed genes involved in cerebral cortex development induced by 0.5 µM of moringin. Heat maps show differentially expressed genes that have Log2FoldChange (LFC) > 2 (red scale) and a q-value (FDR) < 0.05, hence genes that after treatment with 0.5 µM of moringin appear overexpressed and statistically significant. The expression's values in the two conditions, CTR- hPDLSCs and MORINGIN- hPDLSCs, are indicated through the FPKM (Fragments Per Kilobase of exon model per Million mapped fragments) and graphically depicted in green scale. Gene cluster and stage association analyzed by Cortecon database were also shown.

GENE	CTR	MORINGIN 0,5µM	LFC	CLUSTER	STAGE ASSOCIATIONS
**AURKA**	4,69	40,86	3,12	42	Cortical Specifications
**NDC80**	2,39	20,77	3,12	14	Cortical Specifications
**ANLN**	3,71	32	3,11	42	Cortical Specifications
**CDCA2**	1,19	10,17	3,10	42	Cortical Specifications
**NAV2**	0,65	5,39	3,06	35	Upper Layers
**TPX2**	3,78	30,96	3,04	10	Cortical Specifications
**IQGAP3**	1,92	15,67	3,03	14	Cortical Specifications
**HIST1H3D**	49,05	396,7	3,02	40	Neural Differentiation, Cortical Specification, Upper Layers
**CDKN3**	2,66	21,45	3,01	62	Cortical Specification, Upper Layers
**FOXC2**	4,78	38,11	3,00	47	Deep Layers
**SPAG5**	3,12	24,72	2,99	14	Cortical Specifications
**CDC45**	0,59	4,56	2,95	41	Neural Differentiation
**NCAPG**	1,39	10,76	2,95	14	Cortical Specifications
**STIL**	0,98	7,53	2,95	14	Cortical Specifications
**TTK**	1,12	8,65	2,95	14	Cortical Specifications
**HIST1H2AD**	100,28	769,58	2,94	14	Cortical Specifications
**BRCA1**	1,20	9,02	2,92	14	Cortical Specifications
**HIST1H2BN**	88,53	665,05	2,91	12	Cortical Specification, Neural Dfifferentiation
**LMNB1**	2,30	17,02	2,89	14	Cortical Specifications
**DIAPH3**	2,27	16,69	2,88	42	Cortical Specifications
**HIST3H2BB**	3,16	23,03	2,86	63	Cortical Specifications
**CCNB1**	5,72	41,54	2,86	41	Neural Differentiation
**CENPF**	1,89	13,75	2,86	20	Deep Layers
**KIF14**	0,55	4,03	2,86	14	Cortical Specifications
**DEPDC1**	0,42	2,98	2,83	14	Cortical Specifications
**HIST1H2BG**	68,31	478,14	2,81	40	Neural Differentiation, Cortical Specification, Upper Layers
**CENPM**	0,53	3,6	2,77	14	Cortical Specifications
**KIAA0101**	2,29	15,24	2,74	42	Cortical Specifications
**CENPE**	2,08	13,78	2,73	14	Cortical Specifications
**KIF22**	2,45	16,3	2,73	14	Cortical Specifications
**MELK**	2,07	13,64	2,72	42	Cortical Specifications
**BORA**	0,48	3,13	2,71	14	Cortical Specifications
**POLA2**	0,61	3,91	2,69	14	Cortical Specifications
**MAOA**	1,32	8,37	2,66	17	Cortical Specification, Deep and Upper Layers
**ITGA10**	2,78	17,24	2,63	53	Cortical Specification, Upper Layers, Deep Layers

Table S2. Differentially expressed genes involved in cerebral cortex development induced by 0.5 µM of moringin. Heat maps show differentially expressed genes that have Log2FoldChange (LFC) > 2 (red scale) and a q-value (FDR) < 0.05, hence genes that after treatment with 0.5 µM of moringin appear overexpressed and statistically significant. The expression's values in the two conditions, CTR- hPDLSCs and MORINGIN- hPDLSCs, are indicated through the FPKM (Fragments Per Kilobase of exon model per Million mapped fragments) and graphically depicted in green scale. Gene cluster and stage association analyzed by Cortecon database were also shown.

GENE	CTR	MORINGIN 0,5µM	LFC	CLUSTER	STAGE ASSOCIATIONS
**ASPM**	3,02	18,27	2,60	14	Cortical Specifications
**KIF20A**	2,96	17,9	2,60	14	Cortical Specifications
**C15orf41**	1,24	7,41	2,58	14	Cortical Specifications
**BIRC5**	0,75	4,45	2,56	14	Cortical Specifications
**HIST1H2BD**	210,01	1212,03	2,53	11	Deep Layers
**WISP1**	1,15	6,62	2,52	24	Cortical Specification, Upper and Deep Layers
**INCENP**	2,66	15,22	2,52	14	Cortical Specifications
**CRISPLD2**	1,55	8,83	2,51	13	Neural Differentiation and Upper Layers
**KIF20B**	0,94	5,26	2,49	42	Cortical Specifications
**TIMELESS**	1,43	7,98	2,48	42	Cortical Specifications
**MMD**	0,52	2,86	2,46	37	Neural Differentiation
**CDK2**	1,89	10,29	2,45	10	Cortical Specifications
**CMTM1**	0,62	3,3	2,41	30	Cortical Specification, Upper and Deep Layers
**CCNB2**	3,11	16,34	2,39	42	Cortical Specifications
**RIMS1**	0,58	3,01	2,39	42	Cortical Specifications
**FANCI**	5,59	29,23	2,39	14	Cortical Specifications
**ARHGAP11 A**	5,84	30,1	2,37	42	Cortical Specifications
**KIAA1524**	0,96	4,99	2,37	42	Cortical Specifications
**KIF11**	2,47	12,74	2,37	14	Cortical Specifications
**PRC1**	5,85	29,99	2,36	45	Cortical Specification, Upper and Deep Layers
**RACGAP1**	5,39	27,04	2,33	14	Cortical Specifications
**IGF2**	1,13	5,59	2,31	30	Cortical Specification, Upper and Deep Layers
**DHFR**	1,68	8,34	2,31	10	Cortical Specifications
**NCAPG2**	2,36	11,66	2,30	42	Cortical Specifications
**TMPO**	4,46	21,97	2,30	25	Neural Differentiation, Deep Layers
**CKS1B**	5,01	24,52	2,29	14	Cortical Specifications
**HMGB2**	8,27	40,19	2,28	37	Neural Differentiation
**CCDC69**	0,90	4,22	2,23	42	Cortical Specifications
**KPNA2**	28,83	135,67	2,23	42	Cortical Specifications
**CDC7**	0,70	3,26	2,22	42	Cortical Specifications
**FEN1**	1,25	5,81	2,22	42	Cortical Specifications
**CKAP2**	3,86	18,02	2,22	14	Cortical Specifications
**SYBU**	1,07	4,99	2,22	14	Cortical Specifications
**CKS2**	7,61	35,02	2,20	42	Cortical Specifications
**FAM64A**	1,17	5,36	2,20	14	Cortical Specifications

Table S3. Differentially expressed genes involved in cerebral cortex development induced by 0.5 µM of moringin. Heat maps show differentially expressed genes that have Log2FoldChange (LFC) > 2 (red scale) and a q-value (FDR) < 0.05, hence genes that after treatment with 0.5 µM of moringin appear overexpressed and statistically significant. The expression's values in the two conditions, CTR- hPDLSCs and MORINGIN- hPDLSCs, are indicated through the FPKM (Fragments Per Kilobase of exon model per Million mapped fragments) and graphically depicted in green scale. Gene cluster and stage association analyzed by Cortecon database were also shown.

GENE	CTR	MORINGIN 0,5µM	LFC	CLUSTER	STAGE ASSOCIATIONS
**HIST2H2BF**	311,67	1414,09	2,18	45	Cortical Specification, Upper and Deep Layers
**CENPN**	1,76	7,93	2,17	42	Cortical Specifications
**TUBA1C**	203,37	905,9	2,16	63	Cortical Specifications
**CENPA**	1,07	4,76	2,16	14	Cortical Specifications
**DKK1**	0,82	3,65	2,16	13	Neural Diffrentiation, Upper Layers
**TYMS**	8,77	39,22	2,16	10	Cortical Specifications
**NEIL3**	0,95	4,19	2,14	42	Cortical Specifications
**C11orf70**	2,26	9,8	2,12	38	Neural Differentiation, Cortical Specification, Upper and Deep Layers
**CCBL1**	0,76	3,29	2,11	42	Cortical Specifications
**CEBPD**	1,00	4,28	2,10	14	Cortical Specifications
**PCDH9**	0,57	2,44	2,09	10	Cortical Specifications
**PORCN**	8,80	36,95	2,07	42	Cortical Specifications
**PLCE1**	2,12	8,8	2,06	33	Deep Layers
**FBF1**	0,79	3,26	2,05	49	Neural Differentiation, Cortical Specification, Deep Layers
**PSMC3IP**	5,89	23,89	2,02	10	Cortical Specifications
**RFC2**	2,04	8,16	2,00	20	Deep Layers

Table S4. Differentially expressed genes involved in cerebral cortex development induced by 0.5 µM of moringin. Heat maps show differentially expressed genes that have Log2FoldChange (LFC) > 2 (red scale) and a q-value (FDR) < 0.05, hence genes that after treatment with 0.5 µM of moringin appear overexpressed and statistically significant. The expression's values in the two conditions, CTR- hPDLSCs and MORINGIN- hPDLSCs, are indicated through the FPKM (Fragments Per Kilobase of exon model per Million mapped fragments) and graphically depicted in green scale. Gene cluster and stage association analyzed by Cortecon database were also shown.

GENE	CTR	MORINGIN 0,25 µM	LFC	Stat. Significant	CLUSTER	STAGE ASSOCIATIONS
TACC3	2,08	4,81607	1,21	yes	1	Cortical Specification, Upper Layers
GADD45B	0,45	2,75547	2,61	no	2	Neural Differentiation, Cortical Specification, Upper and Deep Layers
HIST1H2AG	116,46	205,385	0,82	yes	2	Neural Differentiation, Cortical Specification, Upper and Deep Layers
HIST1H3J	9,54	16,3133	0,77	no	2	Neural Differentiation, Cortical Specification, Upper and Deep Layers
HIST1H3H	33,03	48,3742	0,55	no	8	Cortical Specification, Upper Layers
PLK4	0,15	2,016	3,75	no	8	Cortical Specification, Upper Layers
CDK2	1,89	6,02959	1,67	yes	10	Cortical Specifications
DHFR	1,68	4,75295	1,50	yes	10	Cortical Specifications
PCDH9	0,57	0,347581	-0,71	no	10	Cortical Specifications
PSMC3IP	5,89	16,3018	1,47	yes	10	Cortical Specifications
TPX2	3,78	13,522	1,84	yes	10	Cortical Specifications
TYMS	8,77	16,4415	0,91	yes	10	Cortical Specifications
HIST1H2BB	37,98	43,3969	0,19	no	11	Deep Layers
HIST1H2BD	210,01	116,148	-0,85	yes	11	Deep Layers
HIST1H2BF	100,56	104,881	0,06	no	11	Deep Layers
HIST1H2BN	88,53	111,179	0,33	no	12	Cortical Specification, Neural Dfifferentiation
CRISPLD2	1,55	8,80879	2,51	yes	13	Neural Differentiation and Upper Layers
DKK1	0,82	11,9212	3,86	yes	13	Neural Diffrentiation, Upper Layers
ASPM	3,02	4,32537	0,52	yes	14	Cortical Specifications
AURKB	0,51	1,96972	1,95	no	14	Cortical Specifications
BIRC5	0,75	3,57498	2,25	yes	14	Cortical Specifications
BORA	0,48	1,64764	1,78	no	14	Cortical Specifications
BRCA1	1,20	2,50354	1,06	no	14	Cortical Specifications
C15orf41	1,24	2,17024	0,81	no	14	Cortical Specifications
CDC20	3,38	9,65262	1,51	yes	14	Cortical Specifications
CDCA5	0,11	0,344472	1,65	no	14	Cortical Specifications
CDCA8	0,48	3,39988	2,82	no	14	Cortical Specifications
CDK1	1,38	4,99573	1,86	yes	14	Cortical Specifications
CEBPD	1,00	3,45673	1,79	no	14	Cortical Specifications
CENPA	1,07	0,988851	-0,11	no	14	Cortical Specifications
CENPE	2,08	4,56014	1,13	yes	14	Cortical Specifications
CENPM	0,53	3,54952	2,74	no	14	Cortical Specifications
CKAP2	3,86	12,318	1,67	yes	14	Cortical Specifications
CKAP2L	0,34	1,34454	1,98	no	14	Cortical Specifications
CKS1B	5,01	27,057	2,43	yes	14	Cortical Specifications
DEPDC1	0,42	1,96926	2,23	no	14	Cortical Specifications
DLGAP5	1,97	5,43013	1,46	yes	14	Cortical Specifications

Table S5. Differentially expressed genes involved in cerebral cortex development induced by 0.25 µM of moringin. Heat maps show differentially expressed genes with Log2FoldChange (LFC) (red scale) and a q-value (FDR), hence genes that after treatment with 0.25 µM of moringin appear overexpressed and statistically significant. The expression's values in the two conditions, CTR- hPDLSCs and MORINGIN-hPDLSCs, are indicated through the FPKM (Fragments Per Kilobase of exon model per Million mapped fragments) and graphically depicted in green scale. Gene cluster and stage association analyzed by Cortecon database were also shown

GENE	CTR	MORINGIN 0,25 µM	LFC	Stat. Significant	CLUSTER	STAGE ASSOCIATIONS
FAM64A	1,17	1,20782	0,05	no	14	Cortical Specifications
FANCI	5,59	13,1129	1,23	yes	14	Cortical Specifications
FBXO5	0,69	2,37136	1,78	no	14	Cortical Specifications
H2AFX	6,02	10,2035	0,76	yes	14	Cortical Specifications
HIST1H2AD	100,28	34,8097	-1,53	yes	14	Cortical Specifications
HJURP	1,40	6,58742	2,23	yes	14	Cortical Specifications
INCENP	2,66	10,4293	1,97	yes	14	Cortical Specifications
IQGAP3	1,92	4,43442	1,21	yes	14	Cortical Specifications
KIF11	2,47	3,45904	0,49	no	14	Cortical Specifications
KIF14	0,55	0,465997	-0,24	no	14	Cortical Specifications
KIF20A	2,96	10,2898	1,80	yes	14	Cortical Specifications
KIF22	2,45	4,2518	0,80	no	14	Cortical Specifications
KIF2C	1,86	5,70463	1,62	yes	14	Cortical Specifications
KIF4A	1,40	5,64136	2,01	yes	14	Cortical Specifications
KIFC1	1,25	3,27046	1,39	no	14	Cortical Specifications
LMNB1	2,30	3,58703	0,64	no	14	Cortical Specifications
MKI67	7,15	33,4566	2,23	yes	14	Cortical Specifications
NCAPG	1,39	4,53732	1,71	yes	14	Cortical Specifications
NDC80	2,39	6,83292	1,52	yes	14	Cortical Specifications
POLA2	0,61	2,85328	2,23	no	14	Cortical Specifications
PSRC1	0,36	1,41184	1,97	no	14	Cortical Specifications
RACGAP1	5,39	9,14221	0,76	yes	14	Cortical Specifications
RAD51	0,37	2,685	2,86	no	14	Cortical Specifications
SGOL1	0,24	1,6878	2,81	no	14	Cortical Specifications
SPAG5	3,12	7,51403	1,27	yes	14	Cortical Specifications
SPC24	2,32	0,00	-7,86	no	14	Cortical Specifications
SPC25	0,00	0,70591	6,14	no	14	Cortical Specifications
STIL	0,98	3,44439	1,81	no	14	Cortical Specifications
SYBU	1,07	1,32057	0,30	no	14	Cortical Specifications
TOP2A	6,35	18,8366	1,57	yes	14	Cortical Specifications
TSC22D3	0,93	0,64743	-0,52	no	14	Cortical Specifications
TTK	1,12	3,64702	1,70	yes	14	Cortical Specifications
UBE2C	3,64	33,0386	3,18	yes	14	Cortical Specifications
ZNF724P	1,44	9,44413	2,71	no	14	Cortical Specifications
MAOA	1,32	3,28999	1,32	no	17	Cortical Specification, Deep and Upper Layers
ZBTB16	0,00	0,00	0,00	no	18	Neural Differentiation, Cortical Specification
CLSPN	0,48	2,85409	2,57	no	19	Neural Differentiation and Upper Layers

Table S6. Differentially expressed genes involved in cerebral cortex development induced by 0.25 µM of moringin. Heat maps show differentially expressed genes with Log2FoldChange (LFC) (red scale) and a q-value (FDR), hence genes that after treatment with 0.25 µM of moringin appear overexpressed and statistically significant. The expression's values in the two conditions, CTR- hPDLSCs and MORINGIN-hPDLSCs, are indicated through the FPKM (Fragments Per Kilobase of exon model per Million mapped fragments) and graphically depicted in green scale. Gene cluster and stage association analyzed by Cortecon database were also shown

GENE	CTR	MORINGIN 0,25 µM	LFC	Stat. Significant	CLUSTER	STAGE ASSOCIATIONS
CENPF	1,89	5,88614	1,64	yes	20	Deep Layers
FANCA	0,55	3,20803	2,54	no	20	Deep Layers
RFC2	2,04	8,16855	2,00	yes	20	Deep Layers
GAR1	0,32	3,46837	3,44	no	24	Cortical Specification, Upper and Deep Layers
WISP1	1,15	0,522156	-1,14	no	24	Cortical Specification, Upper and Deep Layers
TMPO	4,46	8,37091	0,91	yes	25	Neural Differentiation, Deep Layers
HIST1H3G	12,92	110,008	3,09	yes	26	Cortical Specification, Deep Layers
CMTM1	0,62	1,42277	1,20	no	30	Cortical Specification, Upper and Deep Layers
ELN	8,92	0,00	-9,80	yes	30	Cortical Specification, Upper and Deep Layers
IGF2	1,13	1,61749	0,52	no	30	Cortical Specification, Upper and Deep Layers
HIST1H2BE	37,32	77,5545	1,06	yes	33	Deep Layers
PLCE1	2,12	0,980768	-1,11	no	33	Deep Layers
RNASEH2A	6,13	20,5112	1,74	yes	34	Neural Differentiation, Cortical Specification
NAV2	0,65	4,25729	2,71	yes	35	Upper Layers
CEP55	1,24	6,54121	2,40	yes	37	Neural Differentiation
HMGB2	8,27	16,5107	1,00	yes	37	Neural Differentiation
MMD	0,52	1,62304	1,64	no	37	Neural Differentiation
NUSAP1	2,45	8,72113	1,83	yes	37	Neural Differentiation
C11orf70	2,26	3,2644	0,53	no	38	Neural Differentiation, Cortical Specification, Upper and Deep Layers
HIST1H2AE	51,55	142,053	1,46	yes	40	Neural Differentiation, Cortical Specification, Upper Layers
HIST1H2BG	68,31	108,241	0,66	yes	40	Neural Differentiation, Cortical Specification, Upper Layers
HIST1H3B	26,33	130,354	2,31	yes	40	Neural Differentiation, Cortical Specification, Upper Layers
HIST1H3D	49,05	97,2863	0,99	yes	40	Neural Differentiation, Cortical Specification, Upper Layers
CCNB1	5,72	20,9653	1,87	yes	41	Neural Differentiation
CDC45	0,59	1,1472	0,96	no	41	Neural Differentiation
KIF15	0,34	1,3309	1,97	no	41	Neural Differentiation
ANLN	3,71	18,0341	2,28	yes	42	Cortical Specifications
ARHGAP11A	5,84	8,64071	0,57	no	42	Cortical Specifications
AURKA	4,69	27,1687	2,53	yes	42	Cortical Specifications
BRIP1	0,30	1,60486	2,42	no	42	Cortical Specifications
BUB1	1,04	7,66851	2,88	yes	42	Cortical Specifications
BUB1B	2,07	8,92851	2,11	yes	42	Cortical Specifications
C1orf53	0,00	2,43736	7,93	no	42	Cortical Specifications
CCBL1	0,76	1,42157	0,90	no	42	Cortical Specifications
CCDC69	0,90	1,41115	0,65	no	42	Cortical Specifications
CCNA2	1,31	6,4643	2,30	yes	42	Cortical Specifications
CCNB2	3,11	6,91969	1,15	yes	42	Cortical Specifications

Table S7. Differentially expressed genes involved in cerebral cortex development induced by 0.25 µM of moringin. Heat maps show differentially expressed genes with Log2FoldChange (LFC) (red scale) and a q-value (FDR), hence genes that after treatment with 0.25 µM of moringin appear overexpressed and statistically significant. The expression's values in the two conditions, CTR- hPDLSCs and MORINGIN-hPDLSCs, are indicated through the FPKM (Fragments Per Kilobase of exon model per Million mapped fragments) and graphically depicted in green scale. Gene cluster and stage association analyzed by Cortecon database were also shown

GENE	CTR	MORINGIN 0,25 µM	LFC	Stat. Significant	CLUSTER	STAGE ASSOCIATIONS
CDC25C	0,15	1,03751	2,79	no	42	Cortical Specifications
CDC7	0,70	0,549491	-0,35	no	42	Cortical Specifications
CDCA2	1,19	3,03352	1,35	no	42	Cortical Specifications
CENPN	1,76	6,16506	1,81	yes	42	Cortical Specifications
CKS2	7,61	28,0652	1,88	yes	42	Cortical Specifications
DIAPH3	2,27	6,96919	1,62	yes	42	Cortical Specifications
DTL	0,31	1,73	2,48	no	42	Cortical Specifications
ESCO2	0,17	1,42932	3,07	no	42	Cortical Specifications
ESPL1	0,24	0,705713	1,56	no	42	Cortical Specifications
EXO1	0,48	3,56679	2,89	yes	42	Cortical Specifications
FAM83D	1,41	3,48579	1,31	yes	42	Cortical Specifications
FANCD2	1,28	3,27424	1,36	no	42	Cortical Specifications
FEN1	1,25	2,73202	1,13	no	42	Cortical Specifications
HIST1H3E	2,03	2,86956	0,50	no	42	Cortical Specifications
HMMR	0,29	2,71585	3,23	no	42	Cortical Specifications
KIAA0101	2,29	4,74861	1,05	no	42	Cortical Specifications
KIAA1524	0,96	3,23622	1,75	no	42	Cortical Specifications
KIF18B	0,26	1,40759	2,44	no	42	Cortical Specifications
KIF20B	0,94	5,04065	2,42	yes	42	Cortical Specifications
KIF23	1,74	9,90608	2,51	yes	42	Cortical Specifications
KPNA2	28,83	73,3016	1,35	yes	42	Cortical Specifications
MAD2L1	0,64	1,63895	1,36	no	42	Cortical Specifications
MELK	2,07	6,70693	1,70	yes	42	Cortical Specifications
NCAPG2	2,36	6,04363	1,36	yes	42	Cortical Specifications
NEIL3	0,95	2,5996	1,45	no	42	Cortical Specifications
NUF2	0,92	4,71624	2,36	yes	42	Cortical Specifications
PORCN	8,80	23,5098	1,42	yes	42	Cortical Specifications
RAD54L	0,67	2,26452	1,76	no	42	Cortical Specifications
RECQL4	0,22	1,24924	2,51	no	42	Cortical Specifications
RIMS1	0,58	0,00	-5,86	no	42	Cortical Specifications
TIMELESS	1,43	2,8338	0,99	no	42	Cortical Specifications
ZWINT	1,08	4,8393	2,16	yes	42	Cortical Specifications
CASC5	0,74	2,09323	1,50	no	45	Cortical Specification, Upper and Deep Layers
HIST2H2BF	311,67	150,647	-1,05	yes	45	Cortical Specification, Upper and Deep Layers
PRC1	5,85	12,8617	1,14	yes	45	Cortical Specification, Upper and Deep Layers
FOXC2	4,78	5,49017	0,20	no	47	Deep Layers
GTSE1	0,72	1,6898	1,23	no	47	Deep Layers

Table S8. Differentially expressed genes involved in cerebral cortex development induced by 0.25 µM of moringin. Heat maps show differentially expressed genes with Log2FoldChange (LFC) (red scale) and a q-value (FDR), hence genes that after treatment with 0.25 µM of moringin appear overexpressed and statistically significant. The expression's values in the two conditions, CTR- hPDLSCs and MORINGIN-hPDLSCs, are indicated through the FPKM (Fragments Per Kilobase of exon model per Million mapped fragments) and graphically depicted in green scale. Gene cluster and stage association analyzed by Cortecon database were also shown

GENE	CTR	MORINGIN 0,25 µM	LFC	Stat. Significant	CLUSTER	STAGE ASSOCIATIONS
HIST1H3F	0,00	14,1517	10,47	yes	47	Deep Layers
MXD3	0,21	1,28171	2,61	no	47	Deep Layers
FBF1	0,79	0,336546	-1,23	no	49	Neural Differentiation, Cortical Specification, Deep Layers
ITGA10	2,78	0,974451	-1,51	no	53	Cortical Specification, Upper Layers, Deep Layers
HIST1H2BJ	53,78	98,2713	0,87	yes	54	Cortical Specification, Deep Layers
CDKN3	2,66	12,6908	2,25	yes	62	Cortical Specification, Upper Layers
HIST1H4I	12,55	29,4576	1,23	no	62	Cortical Specification, Upper Layers
HIST3H2BB	3,16	10,4037	1,72	no	63	Cortical Specifications
TUBA1C	203,37	770,518	1,92	yes	63	Cortical Specifications

Table S9. Differentially expressed genes involved in cerebral cortex development induced by 0.25 µM of moringin. Heat maps show differentially expressed genes with Log2FoldChange (LFC) (red scale) and a q-value (FDR), hence genes that after treatment with 0.25 µM of moringin appear overexpressed and statistically significant. The expression's values in the two conditions, CTR- hPDLSCs and MORINGIN-hPDLSCs, are indicated through the FPKM (Fragments Per Kilobase of exon model per Million mapped fragments) and graphically depicted in green scale. Gene cluster and stage association analyzed by Cortecon database were also shown

OSTEOGENESIS				
**GENE**	**CTR**	**MORINGIN 0.5 µM**	**LFC**	**Description**
**MYBL2**	1,22	11,37	3,22	MYB proto-oncogene like 2
**E2F1**	0,42	3,33	2,99	E2F transcription factor 1
**BRCA1**	1,20	9,02	2,92	BRCA1, DNA repair associated
**F3**	0,48	3,49	2,87	coagulation factor III, tissue factor
**IGFBP3**	0,98	3,83	1,97	insulin like growth factor binding protein 3
**FZD8**	0,96	3,09	1,68	frizzled class receptor 8
**FOXM1**	4,71	14,00	1,57	forkhead box M1
**PLAT**	17,16	43,29	1,34	plasminogen activator, tissue type
**DUSP1**	12,20	26,65	1,13	dual specificity phosphatase 1
**FOSL1**	8,87	18,61	1,07	FOS like 1, AP-1 transcription factor subunit
**CRYAB**	40,20	82,03	1,03	crystallin alpha B
**INHBA**	7,16	14,02	0,97	inhibin beta A subunit
**TWIST1**	4,47	8,29	0,89	twist family bHLH transcription factor 1
**KLF5**	2,00	3,68	0,88	Kruppel like factor 5
**TGFBR2**	50,16	90,73	0,86	transforming growth factor beta receptor 2
**CTGF**	35,05	59,04	0,75	connective tissue growth factor
**CYBA**	39,78	65,63	0,72	cytochrome b-245 alpha chain
**DEK**	16,06	26,22	0,71	DEK proto-oncogene
**IGFBP5**	17,31	26,97	0,64	insulin like growth factor binding protein 5
**FOS**	66,18	102,20	0,63	Fos proto-oncogene, AP-1 transcription factor subunit
**FASN**	16,30	24,77	0,60	fatty acid synthase
**INSIG1**	4,58	6,87	0,58	insulin induced gene 1
**HSF1**	18,24	27,33	0,58	heat shock transcription factor 1
**PNPLA2**	17,98	26,79	0,58	patatin like phospholipase domain containing 2
**VIMP**	17,56	25,62	0,55	selenoprotein S
**SPARC**	568,80	824,37	0,54	secreted protein acidic and cysteine rich
**FAS**	41,23	58,80	0,51	Fas cell surface death receptor
**PLAUR**	28,92	40,77	0,50	plasminogen activator, urokinase receptor
**GPI**	86,50	118,88	0,46	glucose-6-phosphate isomerase
**TWIST2**	27,31	37,44	0,46	twist family bHLH transcription factor 2
**IL1R1**	13,20	17,81	0,43	interleukin 1 receptor type 1
**NCOR2**	16,40	21,84	0,41	nuclear receptor corepressor 2
**PTPN11**	129,32	170,43	0,40	protein tyrosine phosphatase, non-receptor type 11
**NT5E**	89,56	117,80	0,40	5'-nucleotidase ecto
**CALR**	1305,71	1696,49	0,38	calreticulin
**NCOA3**	13,79	17,70	0,36	nuclear receptor coactivator 3
**MAP2K1**	21,53	27,21	0,34	mitogen-activated protein kinase kinase 1
**NPM1**	426,67	534,43	0,32	nucleophosmin
**SMARCA4**	31,27	38,55	0,30	SWI/SNF related, matrix associated, actin dependent regulator of chromatin, subfamily a, member 4
**GPX1**	232,29	285,87	0,30	glutathione peroxidase 1
**HSPA9**	115,66	140,73	0,28	heat shock protein family A (Hsp70) member 9
**HMGA1**	147,77	178,05	0,27	high mobility group AT-hook 1
**RAC1**	38,09	45,23	0,25	ras-related C3 botulinum toxin substrate 1 (rho family, small GTP binding protein Rac1)
**SUMO3**	26,40	31,23	0,24	small ubiquitin-like modifier 3
**AKT1**	40,37	47,35	0,23	AKT serine/threonine kinase 1
**HTT**	7,31	8,48	0,21	huntingtin
**AXL**	60,67	69,11	0,19	AXL receptor tyrosine kinase
**CDC42**	69,35	78,38	0,18	cell division cycle 42
**THBS1**	985,78	1030,17	0,06	thrombospondin 1
**DCN**	1025,12	1065,48	0,06	decorin
**AKT2**	29,60	26,73	-0,15	AKT serine/threonine kinase 2
**WNT5A**	59,61	53,59	-0,15	Wnt family member 5A

Table S10. Differentially expressed genes involved in osteogenesis induced by 0.5 µM of moringin. In the table genes sorted by decreasing values of Log2FoldChange (LFC) and involved in the pathway of osteogenesis are shown. For each gene expression levels (FPKM) in the two conditions (green scale) and LFC (blue-yellow scale) are reported.

OSTEOGENESIS				
GENE	CTR	MORINGIN 0.5 µM	LFC	Description
CCND1	49,36	43,90	-0,17	cyclin D1
NCOR1	22,00	19,50	-0,17	nuclear receptor corepressor 1
NAMPT	17,61	15,42	-0,19	nicotinamide phosphoribosyltransferase
MAP4K4	110,31	95,53	-0,21	mitogen-activated protein kinase kinase kinase kinase 4
DICER1	7,71	6,63	-0,22	dicer 1, ribonuclease III
SOD1	470,72	404,41	-0,22	superoxide dismutase 1, soluble
CTNNB1	178,84	153,45	-0,22	catenin beta 1
ITGB1	566,52	480,57	-0,24	integrin subunit beta 1
LGALS1	1448,63	1224,51	-0,24	galectin 1
SUMO2	159,43	133,92	-0,25	small ubiquitin-like modifier 2
ITGAV	53,58	44,70	-0,26	integrin subunit alpha V
ACOX1	13,65	11,19	-0,29	acyl-CoA oxidase 1
EIF2AK2	10,23	8,36	-0,29	eukaryotic translation initiation factor 2 alpha kinase 2
WWTR1	10,66	8,39	-0,34	WW domain containing transcription regulator 1
ANGPT1	14,85	11,54	-0,36	angiopoietin 1
MAF	14,38	11,15	-0,37	MAF bZIP transcription factor
ATG7	4,77	3,69	-0,37	autophagy related 7
PRKCE	9,83	7,58	-0,37	protein kinase C epsilon
JAG1	13,69	10,47	-0,39	jagged 1
MGEA5	24,25	18,51	-0,39	meningioma expressed antigen 5 (hyaluronidase)
STAT3	51,37	39,03	-0,40	signal transducer and activator of transcription 3
IFNAR1	5,10	3,86	-0,40	interferon alpha and beta receptor subunit 1
IRF2	9,28	7,01	-0,40	interferon regulatory factor 2
EIF2AK4	41,12	30,96	-0,41	eukaryotic translation initiation factor 2 alpha kinase 4
TCF4	21,79	16,38	-0,41	transcription factor 4
IFRD1	17,29	12,78	-0,44	interferon related developmental regulator 1
CDKN1A	162,96	119,03	-0,45	cyclin dependent kinase inhibitor 1A
LGALS3	201,21	146,90	-0,45	galectin 3
KITLG	9,48	6,88	-0,46	KIT ligand
TRAF3	3,59	2,59	-0,47	TNF receptor associated factor 3
S100A6	1242,85	894,77	-0,47	S100 calcium binding protein A6
MAPK3	37,57	26,56	-0,50	mitogen-activated protein kinase 3
STAT1	188,99	132,26	-0,51	signal transducer and activator of transcription 1
FGFR1	112,67	78,72	-0,52	fibroblast growth factor receptor 1
PDGFC	19,04	13,20	-0,53	platelet derived growth factor C
JUNB	34,68	23,95	-0,53	JunB proto-oncogene, AP-1 transcription factor subunit
CD44	211,36	143,59	-0,56	CD44 molecule (Indian blood group)
HGF	44,17	29,77	-0,57	hepatocyte growth factor
SERPINF1	799,46	527,22	-0,60	serpin family F member 1
SOCS3	7,53	4,79	-0,65	suppressor of cytokine signaling 3
GABBR1	2,58	1,61	-0,68	gamma-aminobutyric acid type B receptor subunit 1
TNFSF12	7,58	4,71	-0,69	tumor necrosis factor superfamily member 12
JUN	38,86	23,87	-0,70	Jun proto-oncogene, AP-1 transcription factor subunit
TFAP2C	12,07	7,35	-0,72	transcription factor AP-2 gamma
SMAD3	17,67	10,53	-0,75	SMAD family member 3
RELB	2,77	1,52	-0,87	RELB proto-oncogene, NF-kB subunit
SMARCD3	8,19	4,38	-0,90	SWI/SNF related, matrix associated, actin dependent regulator of chromatin, subfamily d, member 3
ITGA2	35,82	19,07	-0,91	integrin subunit alpha 2
VEGFA	61,43	29,03	-1,08	vascular endothelial growth factor A
RBPJ	32,99	15,07	-1,13	recombination signal binding protein for immunoglobulin kappa J region
TCF7	3,97	1,68	-1,24	transcription factor 7 (T-cell specific, HMG-box)
ARRB2	4,42	1,87	-1,24	arrestin beta 2
ITGB3	12,75	4,75	-1,42	integrin subunit beta 3
NGF	7,11	2,38	-1,58	nerve growth factor
CHRNA1	14,78	3,63	-2,03	cholinergic receptor nicotinic alpha 1 subunit

Table S11. Differentially expressed genes involved in osteogenesis induced by 0.5 µM of moringin. In the table genes sorted by decreasing values of Log2FoldChange (LFC) and involved in the pathway of osteogenesis are shown. For each gene expression levels (FPKM) in the two conditions (green scale) and LFC (blue-yellow scale) are reported.

ADIPOGENESIS				
GENE	CTR	MORINGIN 0.5 μM	LFC	Description
TSC22D3	0,93	17,51	4,24	TSC22 domain family member 3
KIAA1524	0,96	4,99	2,37	KIAA1524
DKK1	0,82	3,65	2,16	dickkopf WNT signaling pathway inhibitor 1
SLC29A1	2,77	11,28	2,02	solute carrier family 29 member 1 (Augustine blood group)
PTTG1	33,36	119,33	1,84	pituitary tumor-transforming 1
KLF4	7,27	21,17	1,54	Kruppel like factor 4
MYC	4,47	11,64	1,38	v-myc avian myelocytomatosis viral oncogene homolog
HLX	1,89	4,87	1,37	H2.0 like homeobox
NDRG1	43,13	94,96	1,14	N-myc downstream regulated 1
NME1	47,37	103,14	1,12	NME/NM23 nucleoside diphosphate kinase 1
TNC	41,07	87,30	1,09	tenascin C
PIK3R1	9,68	19,45	1,01	phosphoinositide-3-kinase regulatory subunit 1
MIF	78,55	153,56	0,97	macrophage migration inhibitory factor (glycosylation-inhibiting factor)
IRAK1	17,92	31,50	0,81	interleukin 1 receptor associated kinase 1
MET	5,41	9,23	0,77	MET proto-oncogene, receptor tyrosine kinase
MAPK7	4,16	7,00	0,75	mitogen-activated protein kinase 7
HIF1A	57,67	94,20	0,71	hypoxia inducible factor 1 alpha subunit
SHC1	43,73	71,23	0,70	SHC adaptor protein 1
ETS1	2,59	4,07	0,65	ETS proto-oncogene 1, transcription factor
HSPD1	77,68	121,38	0,64	heat shock protein family D (Hsp60) member 1
HRAS	23,59	36,28	0,62	HRas proto-oncogene, GTPase
ADM	8,10	12,30	0,60	adrenomedullin
FN1	4722,61	7065,53	0,58	fibronectin 1
PPIF	4,32	6,30	0,55	peptidylprolyl isomerase F
C5	2,83	4,07	0,52	complement C5
FOXO3	4,08	5,71	0,49	forkhead box O3
FGF7	16,03	22,00	0,46	fibroblast growth factor 7
BCL6	4,40	6,01	0,45	B-cell CLL/lymphoma 6
TGFBR1	5,55	7,52	0,44	transforming growth factor beta receptor 1
CAV1	149,91	200,31	0,42	caveolin 1
FBN1	405,88	516,33	0,35	fibrillin 1
SREBF1	7,63	9,71	0,35	sterol regulatory element binding transcription factor 1
CHUK	9,76	12,40	0,35	conserved helix-loop-helix ubiquitous kinase
MTOR	9,35	11,81	0,34	mechanistic target of rapamycin
PAF1	42,62	51,86	0,28	PAF1 homolog, Paf1/RNA polymerase II complex component
GAPDH	1240,72	1501,53	0,28	glyceraldehyde-3-phosphate dehydrogenase
LEPR	21,84	25,61	0,23	leptin receptor
SMAD4	10,70	12,09	0,18	SMAD family member 4
EGR1	71,99	81,30	0,18	early growth response 1
SREBF2	36,07	32,64	-0,14	sterol regulatory element binding transcription factor 2
SDCBP	84,41	75,22	-0,17	syndecan binding protein
SASH1	16,78	14,82	-0,18	SAM and SH3 domain containing 1
AHR	62,94	55,36	-0,19	aryl hydrocarbon receptor
COL18A1	58,52	51,03	-0,20	collagen type XVIII alpha 1 chain
EPAS1	94,76	82,24	-0,20	endothelial PAS domain protein 1
KDM5B	12,72	10,89	-0,22	lysine demethylase 5B
ERBB2	17,75	14,99	-0,24	erb-b2 receptor tyrosine kinase 2
EIF2AK3	7,62	6,17	-0,30	eukaryotic translation initiation factor 2 alpha kinase 3
NFE2L2	43,18	34,54	-0,32	nuclear factor, erythroid 2 like 2
NRIP1	5,36	4,26	-0,33	nuclear receptor interacting protein 1
SP3	9,36	7,34	-0,35	Sp3 transcription factor
TNFRSF1A	112,14	87,77	-0,35	TNF receptor superfamily member 1A

Table S12. Differentially expressed genes involved in adipogenesis in hPDLSCs treated with 0.5 μM of moringin. In the table genes sorted by decreasing values of Log2FoldChange (LFC) and involved in the pathway of adipogenesis are shown. For each gene expression levels (FPKM) in the two conditions (green scale) and LFC (blue-yellow scale) are reported.

ADIPOGENESIS				
GENE	CTR	MORINGIN 0.5 µM	LFC	Description
ARNT	18,52	14,46	-0,36	aryl hydrocarbon receptor nuclear translocator
CSF1	12,73	9,90	-0,36	colony stimulating factor 1
APP	697,76	537,93	-0,38	amyloid beta precursor protein
BNIP3L	15,53	11,81	-0,40	BCL2 interacting protein 3 like
ITGA1	7,62	5,73	-0,41	integrin subunit alpha 1
IGF1R	16,63	12,47	-0,42	insulin like growth factor 1 receptor
IL6ST	38,01	28,30	-0,43	interleukin 6 signal transducer
IRS1	15,67	11,43	-0,46	insulin receptor substrate 1
MAP3K7	11,88	8,57	-0,47	mitogen-activated protein kinase kinase kinase 7
HMOX1	45,99	32,53	-0,50	heme oxygenase 1
TXNIP	47,93	32,99	-0,54	thioredoxin interacting protein
SAV1	10,94	7,43	-0,56	salvador family WW domain containing protein 1
TLR3	4,60	3,09	-0,57	toll like receptor 3
FHL2	35,06	23,49	-0,58	four and a half LIM domains 2
CAT	144,70	96,69	-0,58	catalase
STAT2	48,00	31,93	-0,59	signal transducer and activator of transcription 2
USF1	9,50	6,31	-0,59	upstream transcription factor 1
NCOA2	9,21	6,09	-0,60	nuclear receptor coactivator 2
RBL2	11,16	7,35	-0,60	RB transcriptional corepressor like 2
MAP2K3	17,18	11,27	-0,61	mitogen-activated protein kinase kinase 3
TFAP2A	20,49	13,32	-0,62	transcription factor AP-2 alpha
SOCS6	2,55	1,64	-0,64	suppressor of cytokine signaling 6
THRB	4,65	2,96	-0,65	thyroid hormone receptor beta
CASP8	12,86	8,18	-0,65	caspase 8
BAX	62,22	39,37	-0,66	BCL2 associated X, apoptosis regulator
PSEN2	10,48	6,55	-0,68	presenilin 2
NRG1	4,37	2,73	-0,68	neuregulin 1
PTEN	43,21	25,81	-0,74	phosphatase and tensin homolog
GJA1	439,36	259,16	-0,76	gap junction protein alpha 1
PPARA	2,46	1,43	-0,78	peroxisome proliferator activated receptor alpha
INSR	2,80	1,55	-0,85	insulin receptor
BCL3	4,56	2,43	-0,91	B-cell CLL/lymphoma 3
DDIT3	8,28	4,38	-0,92	DNA damage inducible transcript 3
SQSTM1	197,31	102,56	-0,94	sequestosome 1
MAPK12	5,86	3,00	-0,97	mitogen-activated protein kinase 12
IRF1	6,63	3,36	-0,98	interferon regulatory factor 1
FLT3LG	11,88	5,98	-0,99	fms related tyrosine kinase 3 ligand
MYD88	14,08	6,82	-1,05	myeloid differentiation primary response 88
WISP2	6,53	2,99	-1,13	WNT1 inducible signaling pathway protein 2
SATB1	12,95	5,84	-1,15	SATB homeobox 1
PTX3	22,15	9,58	-1,21	pentraxin 3
PLAU	45,56	19,44	-1,23	plasminogen activator, urokinase
CASP1	17,76	6,60	-1,43	caspase 1
BDNF	13,31	4,71	-1,50	brain derived neurotrophic factor
NR4A1	5,95	1,84	-1,70	nuclear receptor subfamily 4 group A member 1
THBS4	18,43	5,45	-1,76	thrombospondin 4
PTHLH	8,16	2,19	-1,90	parathyroid hormone like hormone
PGF	31,53	8,10	-1,96	placental growth factor
ABCB4	5,68	1,06	-2,43	ATP binding cassette subfamily B member 4
BMP4	79,33	13,66	-2,54	bone morphogenetic protein 4
CXCL12	69,94	11,81	-2,57	C-X-C motif chemokine ligand 12

Table S13. Differentially expressed genes involved in adipogenesis in hPDLSCs treated with 0.5 µM of moringin. In the table genes sorted by decreasing values of Log2FoldChange (LFC) and involved in the pathway of adipogenesis are shown. For each gene expression levels (FPKM) in the two conditions (green scale) and LFC (blue-yellow scale) are reported.

GENE	CTR	MORINGIN 0.5 µM	LFC	ANNOTATED TERM
ZBTB16	0,00	10,20	13,32	positive regulation of ossification
FOXC2	4,78	38,11	3,00	ossification
IGF2	1,13	5,59	2,31	ossification
DKK1	0,82	3,65	2,16	negative regulation of ossification
CEBPD	1,00	4,28	2,10	positive regulation of osteoblast differentiation
NOCT	0,73	2,78	1,92	negative regulation of osteoblast differentiation
PAM16	4,06	10,79	1,41	ossification
PTK2B	2,98	7,07	1,25	negative regulation of bone mineralization, negative regulation of ossification, regulation of bone mineralization
RORB	7,38	17,33	1,23	negative regulation of osteoblast differentiation
PEX7	3,68	7,90	1,10	endochondral ossification
TNC	41,07	87,30	1,09	osteoblast differentiation
SMOC1	49,73	103,96	1,06	regulation of osteoblast differentiation
FBN2	118,14	221,48	0,91	positive regulation of bone mineralization, positive regulation of osteoblast differentiation
TWIST1	4,47	8,29	0,89	negative regulation of osteoblast differentiation, ossification, osteoblast differentiation, regulation of bone mineralization
MYBBP1A	2,74	4,96	0,85	osteoblast differentiation
ASF1A	4,42	7,80	0,82	osteoblast differentiation
FOXC1	3,46	6,10	0,82	endochondral ossification, ossification
CBS	31,61	55,00	0,80	endochondral ossification
FZD1	4,41	7,49	0,76	canonical Wnt signaling pathway involved in osteoblast differentiation
CTGF	35,05	59,04	0,75	ossification
FBL	24,74	41,47	0,75	osteoblast differentiation
PHB	36,43	60,76	0,74	osteoblast differentiation
HIF1A	57,67	94,20	0,71	negative regulation of bone mineralization, negative regulation of ossification
CREB3L1	85,79	133,92	0,64	osteoblast differentiation, regulation of ossification
IGFBP5	17,31	26,97	0,64	negative regulation of osteoblast differentiation
HSPG2	32,59	50,51	0,63	endochondral ossification
IARS	82,95	127,12	0,62	osteoblast differentiation
FASN	16,30	24,77	0,60	osteoblast differentiation
ZHX3	2,14	3,24	0,60	positive regulation of osteoblast differentiation
SND1	55,66	80,88	0,54	osteoblast differentiation
SYNCRIP	34,41	49,08	0,51	osteoblast differentiation
ASXL2	5,51	7,85	0,51	positive regulation of bone mineralization involved in bone maturation
RPL38	254,85	360,54	0,50	ossification
HDAC7	10,65	14,67	0,46	negative regulation of osteoblast differentiation
SORT1	5,52	7,60	0,46	ossification
TWIST2	27,31	37,44	0,46	negative regulation of osteoblast differentiation, osteoblast differentiation
RASSF2	17,36	23,41	0,43	ossification, regulation of osteoblast differentiation
ASPN	20,27	27,22	0,43	bone mineralization
RPS15	746,23	976,77	0,39	osteoblast differentiation
SNAI2	23,86	30,66	0,36	regulation of osteoblast differentiation
PDLIM7	17,84	22,43	0,33	ossification, positive regulation of osteoblast differentiation
FAT4	6,19	7,76	0,33	ossification involved in bone maturation
RRAS2	17,32	21,40	0,31	osteoblast differentiation
IMPAD1	7,51	9,20	0,29	endochondral ossification
COL1A1	7497,36	9182,01	0,29	endochondral ossification, intramembranous ossification
ALPL	16,45	20,01	0,28	endochondral ossification, osteoblast differentiation
RBMX	33,70	40,70	0,27	osteoblast differentiation
ATP5B	173,25	206,96	0,26	osteoblast differentiation
AKT1	40,37	47,35	0,23	osteoblast differentiation
PSMC2	26,35	30,81	0,23	osteoblast differentiation
SNRNP200	45,69	53,41	0,23	osteoblast differentiation
DHX9	39,82	46,44	0,22	osteoblast differentiation
H3F3B	31,56	36,05	0,19	osteoblast differentiation
SBDS	56,13	64,05	0,19	bone mineralization
CLIC1	121,29	137,85	0,18	positive regulation of osteoblast differentiation
SMAD4	10,70	12,09	0,18	osteoblast differentiation
HNRNPU	41,66	45,99	0,14	osteoblast differentiation
MRC2	86,02	93,46	0,12	osteoblast differentiation
LMNA	1179,18	1226,76	0,06	positive regulation of osteoblast differentiation

Table S14. Up-regulated differentially expressed genes involved in osteogenesis obtained by GO analysis in hPDLSCs treated with 0.5 µM of moringin. In the table genes sorted by decreasing values of Log2FoldChange (LFC) and involved in the pathway of osteogenesis are shown. For each gene expression levels (FPKM) in the two conditions (green scale) and LFC (blue-yellow scale) are reported.

GENE	CTR	MORINGIN 0.5 µM	LFC	ANNOTATED TERM
RPS11	1955,38	1863,83	-0,07	osteoblast differentiation
GNAS	905,33	856,35	-0,08	endochondral ossification, positive regulation of osteoblast differentiation
WNT5A	59,61	53,59	-0,15	positive regulation of ossification
EXT2	55,44	48,09	-0,21	ossification
HSD17B4	117,74	101,52	-0,21	osteoblast differentiation
CTNNB1	178,84	153,45	-0,22	positive regulation of osteoblast differentiation, regulation of osteoblast differentiation
INPPL1	30,75	26,19	-0,23	endochondral ossification
ADAR	51,00	42,71	-0,26	osteoblast differentiation
CD276	24,84	20,68	-0,26	positive regulation of bone mineralization, positive regulation of osteoblast differentiation
BMPR2	14,11	11,60	-0,28	positive regulation of bone mineralization, positive regulation of ossification
DHX36	16,33	13,27	-0,30	ossification
EIF2AK3	7,62	6,17	-0,30	bone mineralization, ossification
DDX5	119,84	96,88	-0,31	regulation of osteoblast differentiation
MEF2D	7,70	6,22	-0,31	endochondral ossification, osteoblast differentiation
CLTC	97,20	78,31	-0,31	osteoblast differentiation
ERCC2	22,99	18,47	-0,32	bone mineralization
TOB1	22,63	18,06	-0,33	negative regulation of osteoblast differentiation
NBR1	42,99	34,24	-0,33	negative regulation of osteoblast differentiation, regulation of bone mineralization
NPR2	61,30	48,73	-0,33	ossification
BMP1	26,40	20,90	-0,34	ossification
WWTR1	10,66	8,39	-0,34	osteoblast differentiation
CHSY1	24,46	19,21	-0,35	negative regulation of ossification
SIX2	36,71	28,80	-0,35	regulation of ossification
SP3	9,36	7,34	-0,35	ossification
MIA3	12,15	9,51	-0,35	positive regulation of bone mineralization
NIPBL	17,17	13,38	-0,36	positive regulation of ossification
COL6A1	3398,09	2645,42	-0,36	osteoblast differentiation
CSF1	12,73	9,90	-0,36	ossification, regulation of ossification
STC1	8,61	6,68	-0,37	ossification
BMPR1A	29,02	22,35	-0,38	positive regulation of bone mineralization, positive regulation of osteoblast differentiation
TOB2	8,74	6,71	-0,38	positive regulation of ossification
JAG1	13,69	10,47	-0,39	positive regulation of osteoblast differentiation
LTBP3	44,16	33,68	-0,39	negative regulation of bone mineralization
NF1	18,43	13,77	-0,42	osteoblast differentiation
IL6ST	38,01	28,30	-0,43	positive regulation of osteoblast differentiation
SATB2	42,29	31,37	-0,43	osteoblast development
TGFBR3	31,74	23,51	-0,43	osteoblast differentiation
FAM20C	39,65	28,97	-0,45	positive regulation of bone mineralization, positive regulation of osteoblast differentiation
ACVR1	44,39	32,03	-0,47	positive regulation of bone mineralization, positive regulation of osteoblast differentiation, regulation of ossification
MMP14	150,72	108,62	-0,47	endochondral ossification, ossification
MAPK3	37,57	26,56	-0,50	regulation of ossification
FGFR1	112,67	78,72	-0,52	negative regulation of osteoblast differentiation
ECM1	222,54	154,57	-0,53	negative regulation of bone mineralization, ossification, regulation of bone mineralization
HDAC4	3,22	2,23	-0,53	negative regulation of osteoblast differentiation, osteoblast development
JUNB	34,68	23,95	-0,53	osteoblast differentiation
SHOX2	4,02	2,76	-0,54	osteoblast differentiation
GLI3	39,46	26,71	-0,56	positive regulation of osteoblast differentiation
EPHA2	15,56	10,47	-0,57	osteoblast differentiation
CAT	144,70	96,69	-0,58	osteoblast differentiation

Table S15. Down-regulated differentially expressed genes involved in osteogenesis obtained by GO analysis in hPDLSCs treated with 0.5 µM of moringin. In the table genes sorted by decreasing values of Log2FoldChange (LFC) and involved in the pathway of osteogenesis are shown. For each gene expression levels (FPKM) in the two conditions (green scale) and LFC (blue-yellow scale) are reported.

GENE	CTR	MORINGIN 0.5 µM	LFC	ANNOTATED TERM
ITGA11	30,39	20,09	-0,60	osteoblast differentiation
GLA	21,09	13,85	-0,61	bone mineralization
BCOR	5,76	3,75	-0,62	negative regulation of bone mineralization
TFAP2A	20,49	13,32	-0,62	positive regulation of bone mineralization
MMP2	2237,29	1450,83	-0,62	intramembranous ossification
KREMEN1	11,51	7,38	-0,64	negative regulation of ossification
THRB	4,65	2,96	-0,65	positive regulation of ossification
TMEM119	27,30	17,19	-0,67	ossification, osteoblast differentiation
OSR1	14,22	8,90	-0,68	positive regulation of bone mineralization
GABBR1	2,58	1,61	-0,68	osteoblast differentiation
RDH14	13,49	8,36	-0,69	osteoblast differentiation
LRP6	7,38	4,49	-0,72	positive regulation of ossification
TXLNG	2,49	1,50	-0,73	regulation of bone mineralization
HDAC5	7,65	4,57	-0,74	negative regulation of osteoblast differentiation, osteoblast development
SMAD3	17,67	10,53	-0,75	negative regulation of osteoblast differentiation, osteoblast development, osteoblast differentiation
GJA1	439,36	259,16	-0,76	osteoblast differentiation, positive regulation of osteoblast differentiation, regulation of bone mineralization
SLC8A1	4,71	2,63	-0,84	positive regulation of bone mineralization
SBNO2	14,38	7,91	-0,86	bone mineralization, ossification
VCAN	336,81	182,50	-0,88	osteoblast differentiation
IFT80	26,65	14,39	-0,89	osteoblast differentiation
DCHS1	6,81	3,54	-0,94	ossification involved in bone maturation
SFRP1	6,31	3,27	-0,95	negative regulation of ossification, negative regulation of osteoblast differentiation, regulation of ossification
EXT1	55,32	28,62	-0,95	ossification
VEGFA	61,43	29,03	-1,08	positive regulation of osteoblast differentiation
RBPJ	32,99	15,07	-1,13	negative regulation of ossification
LRP5	17,45	7,86	-1,15	negative regulation of osteoblast differentiation
SMO	2,74	1,15	-1,26	ossification, osteoblast differentiation
DHRS3	3,61	1,48	-1,29	regulation of ossification
IFITM1	46,36	18,23	-1,35	ossification
ID3	7,83	3,03	-1,37	negative regulation of osteoblast differentiation
GREM1	71,20	24,20	-1,56	negative regulation of bone mineralization, negative regulation of bone mineralization involved in bone maturation, negative regulation of osteoblast differentiation
PTHLH	8,16	2,19	-1,90	bone mineralization, endochondral ossification, osteoblast development
BMP4	79,33	13,66	-2,54	endochondral ossification, ossification, osteoblast differentiation, positive regulation of bone mineralization, positive regulation of ossification, positive regulation of osteoblast differentiation

Table S16. Down-regulated differentially expressed genes involved in osteogenesis obtained by GO analysis in hPDLSCs treated with 0.5 µM of moringin. In the table genes sorted by decreasing values of Log2FoldChange (LFC) and involved in the pathway of osteogenesis are shown. For each gene expression levels (FPKM) in the two conditions (green scale) and LFC (blue-yellow scale) are reported.

GENE	CTR	MORINGIN 0.5 µM	LFC	ANNOTATED TERM
MEX3C	0,00	10,20	13,32	positive regulation of fat cell differentiation
SNAI2	0,42	3,33	2,99	negative regulation of fat cell differentiation
NCOR2	1,00	4,28	2,10	fat cell differentiation, fat cell differentiation
CCDC85B	0,73	2,78	1,92	positive regulation of fat cell differentiation
ALMS1	7,27	21,17	1,54	fat cell differentiation
LPIN1	1,80	5,17	1,52	positive regulation of fat cell differentiation
CEBPD	1,70	4,21	1,31	negative regulation of fat cell differentiation
FAM120B	9,51	22,63	1,25	negative regulation of fat cell differentiation
PSMB8	12,00	24,42	1,02	regulation of fat cell differentiation
RNASEL	12,06	23,42	0,96	brown fat cell differentiation, fat cell differentiation, positive regulation of brown fat cell differentiation
METRNL	3,05	5,66	0,89	negative regulation of fat cell differentiation, negative regulation of fat cell differentiation
FTO	2,00	3,68	0,88	positive regulation of fat cell differentiation
ARID5B	7,16	10,87	0,60	adipose tissue development, fat pad development
HMGA2	4,58	6,87	0,58	negative regulation of fat cell differentiation
UBB	13,90	20,70	0,57	adipose tissue development
LRP5	5,51	7,85	0,51	positive regulation of fat cell differentiation
ADRM1	13,81	19,48	0,50	brown fat cell differentiation
AKT1	2,65	3,70	0,48	fat cell differentiation
ARNTL	5,52	7,60	0,46	negative regulation of fat cell differentiation
ARRDC3	16,40	21,84	0,41	negative regulation of fat cell differentiation, white fat cell differentiation
ASXL1	3,08	4,09	0,41	fat cell differentiation, regulation of fat cell differentiation
ASXL2	4,25	5,54	0,38	regulation of fat cell differentiation, regulation of fat cell differentiation
BBS4	28,34	36,73	0,37	adipose tissue development
CARM1	21,93	28,42	0,37	negative regulation of fat cell differentiation
CCND1	23,86	30,66	0,36	positive regulation of fat cell differentiation, positive regulation of fat cell differentiation, white fat cell differentiation
CDK4	7,63	9,71	0,35	fat cell differentiation
CMKLR1	9,35	11,81	0,34	regulation of brown fat cell differentiation
CSF1	232,29	285,87	0,30	fat cell differentiation
DDIT3	31,56	38,71	0,29	positive regulation of fat cell differentiation
E2F1	48,94	58,70	0,26	adipose tissue development
EIF2AK3	40,37	47,35	0,23	positive regulation of fat cell differentiation
GPS2	18,81	21,88	0,22	positive regulation of fat cell differentiation
GPX1	16,57	19,15	0,21	adipose tissue development
GRK5	27,03	30,71	0,18	adipose tissue development, regulation of brown fat cell differentiation
IFT88	1179,18	1226,76	0,06	negative regulation of adipose tissue development
INSIG1	59,61	53,59	-0,15	negative regulation of fat cell differentiation
ITGA6	49,36	43,90	-0,17	fat cell differentiation
JAG1	17,11	15,17	-0,17	fat cell differentiation
KLF4	19,03	16,57	-0,20	negative regulation of fat cell differentiation
KLF5	23,38	19,55	-0,26	negative regulation of fat cell differentiation
LAMA4	21,14	17,45	-0,28	fat cell differentiation, fat cell differentiation
LAMB3	7,62	6,17	-0,30	fat cell differentiation
LMNA	10,66	8,39	-0,34	negative regulation of fat cell differentiation
LRP6	4,88	3,81	-0,36	fat cell differentiation
MMP11	17,17	13,38	-0,36	fat cell differentiation
MTOR	12,73	9,90	-0,36	mammary gland fat development
NIPBL	15,20	11,75	-0,37	adipose tissue development
NOCT	13,69	10,47	-0,39	negative regulation of fat cell differentiation
NR1D1	28,51	21,12	-0,43	fat pad development
NR4A1	18,09	13,37	-0,44	fat cell differentiation
NUCB2	6,44	4,71	-0,45	fat cell differentiation
OSBPL11	1415,11	986,83	-0,52	adipose tissue development, fat pad development
OSBPL8	10,94	7,43	-0,56	positive regulation of fat cell differentiation
PER2	69,39	46,37	-0,58	negative regulation of fat cell differentiation
PIK3CA	5,61	3,70	-0,60	fat pad development
PLCB1	64,70	40,44	-0,68	fat cell differentiation, fat cell differentiation
RGS2	5,73	3,55	-0,69	regulation of fat cell differentiation
SAV1	7,38	4,49	-0,72	negative regulation of fat cell differentiation
SELENBP1	3,32	2,02	-0,72	white fat cell differentiation
SFRP1	17,67	10,53	-0,75	negative regulation of fat cell differentiation
SMAD3	17,48	10,29	-0,76	brown fat cell differentiation
SORT1	4,85	2,84	-0,77	fat cell differentiation, fat cell differentiation
SPG20	12,34	6,69	-0,88	brown fat cell differentiation
SREBF1	8,28	4,38	-0,92	negative regulation of fat cell differentiation
TBL1X	6,31	3,27	-0,95	positive regulation of fat cell differentiation
TRIB2	52,26	26,33	-0,99	brown fat cell differentiation
TRIO	61,43	29,03	-1,08	negative regulation of fat cell differentiation
VEGFA	12,33	5,71	-1,11	adipose tissue development, fat pad development, fat cell differentiation
WNT5A	16,50	7,57	-1,12	fat cell differentiation, regulation of fat cell differentiation
WWTR1	17,45	7,86	-1,15	adipose tissue development, adipose tissue development, positive regulation of fat cell differentiation
XBP1	10,03	4,30	-1,22	brown fat cell differentiation
ZBTB16	4,16	1,62	-1,36	negative regulation of fat cell differentiation
ZFP36	4,83	1,53	-1,66	negative regulation of fat cell differentiation
ZFP36L2	5,95	1,84	-1,70	fat cell differentiation

Table S17. Up and down-regulated differentially expressed genes involved in adipogenesis obtained by GO analysis in hPDLSCs treated with 0.5 µM moringin. In the table genes sorted by decreasing values of Log2FoldChange (LFC) and involved in the pathway of adipogenesis are shown. For each gene expression levels (FPKM) in the two conditions (green scale) and LFC (blue-yellow scale) are reported.

ONCOGENES				
GENE	CTR	MORINGIN 0.5 µM	LFC	q_value
BCL11A	0,00	0,12	16,87	1,000
ETV4	0,19	2,12	3,47	1,000
CCND2	0,70	1,03	0,56	1,000
MAFB	0,66	0,78	0,24	1,000
PIM1	1,88	1,97	0,07	1,000
MAML2	8,51	8,87	0,06	0,618
CBLB	12,74	13,12	0,04	0,761
BCL2	0,00	0,00	0,00	1,000
CDX2	0,00	0,00	0,00	1,000
EVI1	0,00	0,00	0,00	1,000
LCK	0,00	0,00	0,00	1,000
MYB	0,00	0,00	0,00	1,000
MYCN	0,00	0,00	0,00	1,000
TCL1A	0,00	0,00	0,00	1,000
USP6	0,00	0,00	0,00	1,000
GOPC	2,90	2,86	-0,02	0,938
SS18	17,10	15,94	-0,10	0,411
BRAF	15,34	14,02	-0,13	0,353
DDX6	20,90	19,03	-0,13	0,075
AKT2	29,60	26,73	-0,15	0,032
BCR	7,38	6,63	-0,15	0,220
ELK4	4,99	4,46	-0,16	0,474
CCND1	49,36	43,90	-0,17	0,003
PLAG1	0,47	0,41	-0,21	1,000
ERBB2	17,75	14,99	-0,24	0,019
MAF	14,38	11,15	-0,37	0,008
MITF	2,60	1,94	-0,42	0,152
SMO	2,74	1,15	-1,26	0,002
KIT	0,58	0,05	-3,68	1,000

TUMOR SUPPRESSOR GENES				
GENE	CTR	MORINGIN 0.5 µM	LFC	q_value
CDH11	42,96	40,85	-0,07	0,14951
CDK6	0,92	1,66	0,86	1,0000
CREB1	2,27	2,01	-0,18	1,0000
FH	43,51	43,20	-0,01	0,92638
GPC3	0,94	0,81	-0,22	1,0000
IL2	0,00	0,00	0,00	1,0000
JAK2	2,72	1,99	-0,45	0,06889
MDM4	1,36	1,12	-0,27	1,0000
MEN1	9,60	11,44	0,25	0,11484
PML	34,45	30,49	-0,18	0,05082
SOCS1	1,03	2,66	1,36	0,10327
SYK	0,00	0,00	0,00	1,0000
TCF3	4,41	4,74	0,10	0,63441
TNFAIP3	0,70	2,06	1,56	1,0000
PALB2	4,07	5,37	0,40	0,04424

Table S18. Oncogenes and Tumor Suppressor genes expression. In the heat maps expression levels (FPKM) (green scale), LFC (red scale) and q_value (blue scale) which corresponds to FDR statistical test (False Discovery Rate) of oncogenes and tumor suppressor genes in the two conditions CTR- hPDLSCs and 0.5 µM MORINGIN- hPDLSCs were described. The resulting values appear, in most cases, not statistically significant. The heat maps indicate that genes belonging to these two groups are not differentially expressed in the two conditions.
